# An integrative review of adult patient-reported reasons for non-urgent use of the emergency department

**DOI:** 10.1186/s12912-023-01251-7

**Published:** 2023-03-30

**Authors:** Amanda McIntyre, Shannon Janzen, Lisa Shepherd, Mickey Kerr, Richard Booth

**Affiliations:** 1 Arthur Labatt Family School of Nursing, University of Western Ontario, London, Canada; 2 Department of Emergency Medicine, University Hospital, London Health Sciences Centre, London, Canada; 3 London, Canada; 4 Schulich School of Medicine and Dentistry, University of Western Ontario, London, Canada

**Keywords:** Nurses, Nursing, Emergency department, Emergency room, Emergency medicine, Emergency nursing, Decision-making, Non-urgent use, Perception, Patient

## Abstract

**Objective:**

To conduct an integrative review of the scientific literature to explore adult patient-reported reasons for using the emergency department (ED) non-urgently.

**Method:**

A literature search of CINAHL, Cochrane, Embase, PsycINFO, and MEDLINE was conducted with filters for humans, published January 1, 1990-September 1, 2021, and English language.

Methodological quality was assessed using Critical Appraisal Skills Programme Qualitative Checklist for qualitative and National Institutes Health (NIH) Quality Assessment Tool for Observational Cohort and Cross-Sectional Studies for quantitative studies. Data was abstracted on study and sample characteristics, and themes/reasons for ED use. Cited reasons were coded using thematic analysis.

**Results:**

Ninety-three studies met inclusion criteria. Seven themes were found: need to be risk averse with respect to the health issue; knowledge and awareness of alternative sources of care; dissatisfaction with primary care provider; satisfaction with ED; ED accessibility and convenience resulting in low access burden; referred to the ED by others; and relationships between patients and health care providers.

**Discussion:**

This integrative review examined patient-reported reasons for attending the ED on a non-urgent basis. The results suggest that ED patients are heterogenous and many factors influence their decision-making. Considering the complexity with which patients live, treating them as a single entity may be problematic. Limiting excessive non-urgent visits likely requires a multi-pronged approach.

**Conclusion:**

For many ED patients, they have a very clear problem which needed to be addressed. Future studies should explore psychosocial factors driving decision-making (e.g., health literacy, health-related personal beliefs, stress and coping ability).

**Supplementary Information:**

The online version contains supplementary material available at 10.1186/s12912-023-01251-7.

## Introduction

Internationally, there is increasing patient demand for health care services at accident and emergency departments (ED) [1]. According to Morgans et al. [2] a health emergency is defined as "a sudden or unexpected threat to physical health or wellbeing which requires an urgent assessment and alleviation of symptoms” (p. 288). There is little agreement between clinicians and patients as to what constitutes an *emergency* situation requiring urgent or emergency health care services. In clinical practice, health care providers tend to conceptualize emergencies as those which are structured around physiological metrics that suggest a critical threat to life or a limb (i.e., death or serious injury) [2]. Conversely, researchers have shown that patients commonly minimize, or fail to recognize, medically significant symptoms, and focus instead on the nature of their symptoms [2]. Symptoms which present with severe, sudden, or rapid onset tend to be interpreted as an emergency whereas those which are slow and intermittent are considered less urgent [2].

Despite the original intent of the ED, visits for low acuity reasons are common and have been described as *non-urgent, inappropriate*, *preventable*, *avoidable*, and/or *misuse* in the scientific literature. In an extensive review, the prevalence of non-urgent ED use has been reported to range from 10% to 90% with approximately half of included studies having a non-urgent ED use prevalence rate of 24% to 40% [3]. This may be problematic as research has shown that non-urgent users complicate the provision of medical services, impair treatment for patients with emergent health needs, and make it difficult to properly assess medical acuity [4]. With the goal of fully understanding and addressing this problem, an evaluation of patient-reported reasons for non-urgent ED is required.

### Background

Numerous studies have specifically examined factors influencing use of the ED on a non-urgent basis. Since 2009, five reviews with different methods and foci have captured varying aspects of this literature [3, 5–8]. Carret et al. [3] and Uscher-Pines et al. [8] both conducted a systematic literature review of quantitative research studies (retrospective and prospective) and examined variables (i.e., sociodemographic and clinical factors) associated with non-urgent use. The reviews by Kraaijvanger et al., [7] Coster et al., [6] and O’Cathain et al. [5] included both qualitative and quantitative studies of both adult and pediatric populations; they all specifically explored patient-reported reasons for ED use. Kraaijvanger et al. [7] performed a systematic review and meta-analysis and Coster et al. [6] performed a rapid (non-systematic) review; O’Cathain et al. [5] performed a realist (non-systematic) review, expanding on these two reviews.

While the academic literature contains various syntheses related to the subject of non-urgent ED use, limitations exist in terms of the 1) heterogeneity of the included population, 2) type of methods employed (systematic versus non-systematic literature searching), 3) assessment of quality and completeness of data culling and abstraction, and 4) subjective reporting of reasons for non-urgent ED use (i.e., patient reported versus inferred). For example, inclusion criteria varied significantly between reviews; the pediatric population was included in three of the five reviews, [5–7] patients arriving at the ED via ambulance were included in one review, [5] and specific disease categories were included for two reviews [3, 7]. Combining these different populations is problematic as there are clear differences in the decision-making process for medical care of children by parents and caregivers, as well as for taking an ambulance ride (versus walking in the front door). Non-systematic methods were used in two reviews [5, 6] and another review [8] included only American articles. It was not always clear in the reviews how patients were triaged “non-urgent.” Assessments of methodological quality were only reported by Coster et al. [6]. In some reviews, tabled data and reference lists were incomplete. Given the heterogeneity of methods, design, quality appraisal of resources, and synthesis approach, it is difficult to draw meaningful conclusions on patient-reported reasons for ED use. A comprehensive review with defined criteria may better inform practice and policy moving forward.

## The review

### Objective

The objective of this review was to conduct an integrative review of the scientific literature to explore adult patient-reported reasons for using the ED non-urgently.

### Design

A study protocol was not previously registered for this review.

An integrative review of the evidence was performed using the methodology described by Whittemore and Knafl [9]. An integrative review is a review method which summarizes empirical or theoretical literature in an effort to comprehensively understand phenomena or a health care problem [10]. They are often used in nursing science where a review of the state of science may directly inform research, practice and policy [9]. The integrative review method allows for the inclusion of a number of different methodologies (i.e., experimental, non-experimental, qualitative, and quantitative) and therefore may be applicable to problems of importance in nursing [9]. Given the nature of the topic, as well as the heterogeneity of design types, this method was deemed most appropriate. The five-stage review approach by Whittemore and Knafl [9] was undertaken: (1) problem identification (i.e., introduction, background, aim), (2) literature search, (3) data evaluation, (4) data analysis, and (5) presentation.

### Search strategy

A literature search of multiple databases (i.e., CINAHL, Cochrane, Embase, PsycINFO, MEDLINE, and Scopus) was conducted by applying a systematic approach consistent with the Preferred Reporting Items for Systematic Reviews and Meta-Analyses (PRISMA). The search strategy was developed in an iterative form in consultation with a health sciences librarian. It involved three overarching constructs related to the ED, non-urgent care, and decision-making, with MeSH terms, key words, and subject headings used for each database, as appropriate. Filters were applied for the following restrictions: studies involving humans, published between January 1, 1990 and September 1, 2021, and in the English language. Supplementary search techniques consisted of scanning the reference lists of retrieved articles and reviews on the topic for missed citations.

Articles retrieved from each database search were downloaded to EndNote (Version 9.0). After removing duplicates, each article title was assessed for relevance by two screeners (AMc, SJ). Relevant abstracts and subsequent full-text articles were then screened according to the following four [4] *a priori* inclusion criteria:

1. Patients were adults (mean age = 18 years of age or older);
2. Patients were recruited prospectively from an ED, also commonly referred to as the emergency room, accident and emergency care, or accident and emergency department;
3. Patients were specifically asked for their reason for seeking emergency care services;
4. Using any method, patients were triaged on the basis of the severity and urgency of their presenting condition or reason for visit.

The following types of studies were excluded: general, non-systematic reviews, expository/textbook chapters, conference proceedings, program reviews/descriptions (without a study sample), continuous learning/education modules, and clinical practice guidelines. If a study included a sample with only one specific medical condition or disease (e.g., asthma or congestive heart failure or epilepsy), or the study examined reasons for taking an ambulance to the ED, it was excluded. Studies assessing frequent, repeat or high-use ED users only were excluded. There was no minimum sample size required for inclusion.

### Quality appraisal

Two independent reviewers (AMc, SJ) assessed each study for methodological quality using two commonly used quality assessment tools. Qualitative studies were assessed using the Critical Appraisal Skills Programme Qualitative Checklist (CASP) [11]. The CASP is a 10-item questionnaire which allows one to evaluate qualitative studies among three broad areas: 1) Are the results of the study valid (Section A); 2) What are the results (Section B); and 3) Will the results help locally? (Section C). Items were rated either ‘yes’ or ‘not reported’ (i.e., not reported or could not tell). The CASP authors do not suggest scoring the items. Quantitative studies were assessed using the National Institutes Health (NIH) Quality Assessment Tool for Observational Cohort and Cross-Sectional Studies [12]. This tool includes 14 items evaluating a wide range of quality measures; however, some items are more relevant to cohort studies (items 6–10, 12, 13). We defined quantitative, cross-sectional studies as those using methods such as in-person or postal surveys and/or structured interviews where statistical analysis was performed. The CASP and NIH tools have been previously used to evaluate studies on non-urgent use of the ED in a previous rapid review by Coster et al. [6].

### Data abstraction

Two reviewers (AMc, SJ) abstracted the following data from each study included for review: author(s), year of publication, country of first author’s origin, study design (i.e., quantitative, qualitative, mixed methods, or review), sample size, method of triage (i.e., triage system, definition or list of explicit criteria), patient characteristics (e.g., age, gender), study aim/objective, data collection method, and themes/reasons for ED use. The reasons for ED use were abstracted in the manner and language in which they were reported by the original authors. Data were abstracted and summarized in tabular form.

### Synthesis

Whittemore and Knafl [9] describe the importance of identifying themes in the data abstraction and synthesis process. As such, the two reviewers coded and identified themes (data analysis stage) from each of the study’s key results. A structured, six-phase thematic analysis was applied using the approach by Braun and Clark [13] (Table 1). This method can be adapted for different types of data (including reviews) [14]. This approach has recently been used for assessing both qualitative and quantitative studies in an integrative review [15]. Specific data synthesis actions are outlined in Table 1. For qualitative studies, the original themes identified by individual studies (with supporting quotes and examples) were reviewed, coded and iteratively compared until large overarching themes between studies were uncovered. For quantitative studies, authors primarily reported results in tabular or list format whereby patient reported reasons were given (usually as statements), along with the proportion of the sample reporting this reason. Similar to qualitative articles, the list of reasons reported by quantitative studies were reviewed iteratively, coded and compared between studies until overarching themes emerged.

**Table 1:** Braun and Clarke’s [13] phases of thematic analysis, as adapted by Cooper et al. [15], and the research teams’ data synthesis actions

**Phase**		**Action**
**1**	Familiarizing yourself with the data	• Each included study was re-read following the quality appraisal process • Each study’s key findings were abstracted verbatim and displayed in a display tables (Tables 2–3)
**2**	Generating initial codes	• Initial codes were generated and applied to *each* finding in the display table • Each unique code was placed in a code table (table not shown)
**3**	Searching for themes	• In an iterative manner, similarities of concepts were explored among the various codes assigned • Codes were gradually grouped together within preliminary themes
**4**	Reviewing themes	• Preliminary themes were then compared and contrasted to examine similarities and differences • Re-coding was performed as necessary • Themes were discussed by researchers to reach consensus
**5**	Defining and naming themes	• Themes were named and a detailed description of each theme was drafted (scope, breadth, depth), including the use of study examples
**6**	Producing the report	• Findings written up with supporting evidence of themes within the data

## Results

### Included studies

**Fig. 1:**
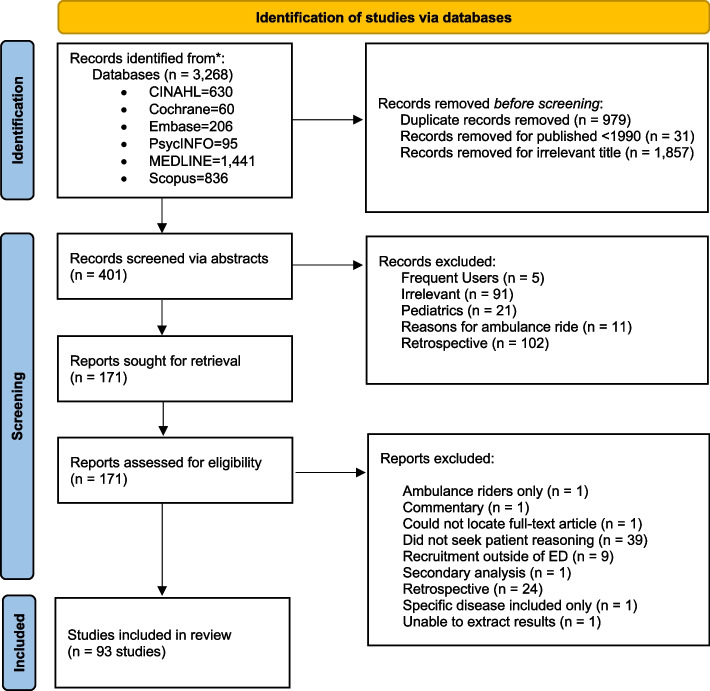
PRISMA flow diagram for the systematic literature search

The literature review returned 3,268 studies; 401 abstracts and 171 full-length texts were reviewed, from which a total of 93 studies met the previously stated inclusion criteria (Fig. 1).

Among the 93 articles included, 69 were quantitative studies [16–84], 21 were qualitative studies [85–105] and 3 were reviews [5–7] (Tables 2 and 3). CASP and NIH quality criteria were applied to included articles to consider their rigour. Articles were not excluded based on rigour, rather, areas where there were concerns about rigour within an article were then discussed amongst the research team and themes did not rely solely on such articles. Overall, the included articles were shown to be of very high quality.

**Table 2:** Study and sample characteristics, quality appraisal score, and key themes/identified reasons for emergency department use among *qualitative* studies

**Study Characteristics** **Data Collection** **Sample Size** **CASP Score**	**Sample Characteristics** **Formal Triage Method**	**Key Themes/Issues Identified for Reasons for Use**
(Bornais, Crawley et al. 2020) [100] Canada Qualitative Study, Semi-structured Interview *N* = 33 (30 patients, 3 caregivers) CASP = 10	Mean age: 40.3 ± 17.3 yr (19 to 72 yr); Gender: males = 12, females = 21 Triage: Not specified (included patients determined by an ED nurse as “non-urgent”)	• Practitioner referral • Primary care provider was contacted first but referred to ED instead for care (*N* = 12) • Efficacy of care (*N* = 16) • Everything “needed” is in one spot • Leave with an answer • Access to specialists • Time Saver (*N* = 26) • Waiting for primary care provider could take days/weeks • All testing done same day
(Claver 2011) [86] United States Qualitative Study, Semi-structured interviews *N* = 30 CASP = 10	Mean age: 79.3 ± 8.2 yr (56 to 92 yr); Gender: males = 30, females = 0 Triage: Not specified	• *Illness Burden*—Those with high illness burden felt no choice in decision to do to ER, often told to by someone else, severity of symptoms was a factor • *Knowledge* – influenced by knowledge about the course of their chronic illness and acute flare-ups, past experience with ER and potential care ER could provide • *Insufficient self-care* – attempts at self-care/treatment at home is not working, most spoke of a wait-and-see method
(Durand, Palazzolo et al. 2012) [93] France Qualitative: Semi-structured interviews *N* = 87 CASP = 10	Mean age: 38.3 yr ± 16.2 yr [17–78]; Gender: males = 47, females = 40 Triage: Not specified (determined by the nurse whether the presenting complaint could be taken care of by a primary care physician (non-urgent) or not (urgent))	• Fulfil health care needs (35.6%) and anxiety generated by the complaint (29.9%), as well as to relieve pain • Barriers to primary care providers (e.g., difficulty obtaining appointment, difficulty accommodating their work schedules, understood options and alternatives and made choice) • Advantages of the ED (e.g., availability of resources, diagnostic tests and treatment, availability of availability of medication, cared for in a single location)
(Long, Knowles et al. 2021) UK [88] Qualitative interviews *N* = 16 (*N* = 8 for ED) CASP = 10	Mean age: 25 yr (18–30 yr); Gender: males = 5, females = 11 Triage: Not specified (in each service, clinicians identified patients they considered had made ‘clinically unnecessary’ use of the service; that is, the patient could have used a lower acuity service or self-care)	Results are pooled for all three settings (i.e., ED results not stratified) • *Concern about the seriousness of symptoms and desire for reassurance* – feelings of anxiety and unfamiliarity were large triggers for both psychological and physical symptoms • *Reduced coping capacity due to poor mental health, stress, lack of resources* – need for immediate relief (especially for pain), inability to cope due to stressful lives • *Influence of others* – influence of others in social networks, perceptions or prior experiences of services (other peoples) • *Concern about the impact of symptoms on daily life* – concerned, unable to access GP quickly enough • *Positive and negative views of different services* • *Frustration at lack of resolution of an on-going problem, despite previous efforts –* waiting long enough for things to improve/resolve
(Gomide MF 2012) [101] Brazil Qualitative: interviews *N* = 23 CASP = 10	Mean age: 40 yr; Gender: males = 10, females = 13 Triage: Not specified	• Difficulty getting immediate care at other services • Limited hours of primary care • Limited time to primary care due to work obligations • EDs have more diagnostic resources
(Goodridge and Stempien 2019) [98] Canada Qualitative Study, Semi-structured interviews *N* = 115 (family member accompanied participant in 72 cases) CASP = 10	Mean age: 79.1 yr (65–98 yr); Gender: males = 47, females = 68 Triage: Canadian Triage and Acuity Scale (CTAS)	• Referred by GP or specialist (*N* = 36) • GP was not available (*N* = 3) • *Accessibility*—Ease of access to comprehensive medical, diagnostic and multidisciplinary services in one location. Felt they had exhausted their own repertoire of solutions and needed help to manage issue • *Availability* – Only option after business hours • *Quality of Care* – thought care quality was superior in ED and offered better continuity of care if they had a complex medical history (e.g., access to tests, treatments, admissions) • *Previous Experience* – having tried to access primary care first in the past and being referred to ED influenced decision
(Guttman, Zimmerman et al. 2003) [94] USA Structured interview with open-ended questions *N* = 77 CASP = 10	Age: 19–25 yr = 11, 26–35 yr = 22, 36–45 yr = 12, 46–55 yr = 8; Gender: males = 41, females = 36 Triage: Not specified (considered “non-urgent” by ED triage staff)	• Conceptions of needs (e.g., relief of pain/discomfort, reassurance, approval/second opinion, treatment, advice, financial) • Conceptions of appropriateness (e.g., causing concern, after-hours services, unavailability/issues with primary care) • Preference (e.g., geographical proximity, familiarity, trust, shorter wait, resources/facilities/staff availability, one-stop)
(Henninger, Spencer et al. 2019) [102] Switzerland Qualitative, Semi-structured interviews *N* = 20 (GP: *N* = 9; ED: *N* = 11) CASP = 10	Mean age: 44.2 ± 34.6 yr (19 to 82 yr); Gender: males = 9, females = 11 Triage: Swiss Emergency Triage Scale (SETS)	Factors influencing decision where to consult (GP or ED): • *Relationship with GP*—Those with strong relationships/trust in GP went first to GP, patients liked continuity of care offered by GP • *Perceived nature of the complaint—*Chest pain and severe headaches were reason to consult ED • *Anticipated wait time before being seen*—Those needing care out of office hours more likely to use ED, rapid answers given by ED appealing to some, booking appointment with GP reduces “wait time” in waiting room Strong themes in favour of attending ED: • Technical equipment (e.g., radiology) • Open hours (24/7) • Access to specialists
(Howard, Davis et al. 2005) [99] USA Qualitative: Structured interview with open-ended questions *N* = 31 CASP = 10	Mean age: 34 yr (22–43 yr); Gender: not reported Triage: Standards set by Kentucky Emergency Nurses Association	• They were unable to obtain an appointment with a PCP (e.g., clinic not open, too late for a reply, unable to get in that day) • They were referred by the staff (not the doctor) in PCP’s offices to be evaluated in the ED • It took less of their time to be seen in the ED than it did to contact their PCP, only to be told to go to the ED
(Keizer Beache and Guell 2016) [87] St Vincent and the Grenadines, Caribbean Grounded theory approach: Semi-structured interviews *N* = 12 CASP = 10	Age: 19–72 yr; Gender: males = 7, females = 5 Triage: Not specified (“Non-urgent” status determined by triage nurse)	• Habitual use of the ED (i.e., automatic/habitual behaviour; difficulty answering questions (short phrases) regarding roles/functions of AED, unable to differentiate between the roles of AED and district clinics, widely shared practice, socially encouraged) • Health system (private and public) encouraged or initiated use of AED (i.e., clinic schedule, type of staff/doctor seeking, belief that district clinic staff refers patient to AED, dissatisfaction with the behaviour of clinic staff, free service at AED) • Deliberate use of AED (i.e., convenience, based on patients’ assessed seriousness of their complaint, past positive AED experiences, confidence in AED, no cost, familiarity with AED)
(Koziol-McLain, Price et al. 2000) [96] USA Narrative Descriptive: Unstructured interviews *N* = 30 CASP = 10	Mean age: 31 yr (17–60 yr); Gender: males = 8, females = 22 Triage: Not specified (4-level triage system from 1 (life-threatening) to 4; patients included if triage level 2–4)	• Toughing it out (i.e., dealing with the issue before going to ED) • Symptoms overwhelming self-care measures (i.e., use of over-the-counter medicines not working, medical issue impacts functioning) • Calling a friend (i.e., social support/advice from friends, relatives, particularly maternal figure) • Nowhere else to go (i.e., could not access alternative medical services, referred to ED by other healthcare providers) • Convenience (i.e., work schedules, child care, transportation)
(Kraaijvanger, Rijpsma et al. 2017) [95] Netherlands Qualitative Study, Structured interview *N* = 30 CASP = 10	Mean age: 46 yr; Gender: males = 19, females = 11 Triage: Manchester Triage System (MTS)	Health Concerns • Anxiety about presenting symptoms and consequences of being left untreated • Expecting to need secondary care and wanted access to additional investigations/testing/treatment that are not provided by GP • Receiving treatment in hospital for the presenting condition already Practical Issues • Perceived easier accessibility of the ED (no appointments needed, always accessible, no restrictions, more timely appointments than waiting for GP) • Distance – not from the area and unfamiliar with where else to access care. Others from the area were closer to ED
(Matifary, Wachira et al. 2021) [97] Kenya Qualitative Study, Semi-structured interviews *N* = 24 CASP = 10	Mean age: 31.8 ± 8.8 yr (25 to 55 yr); Gender: males = 12, females = 12 Triage: Canadian Triage and Acuity Scale (CTAS)	• Feel unwell, want answers to why they are feeling unwell • Positive experience in the past (efficient care, satisfied with services provided and quality of care) • Other services closed • Influenced by media in the form of advertisements Some participants just needed a way to access care
(McKenna, Rogers et al. 2020) [104] UK Semi-structured interviews, social network mapping *N* = 40 (Demographics *N* = 34) CASP = 10	Age: 20–40 yr = 14; 40–60 yr = 11, 60–80 yr = 8, > 80 yr = 1; Gender: males = 14, females = 20 Triage: Not specified (included all participants triaged on arrival as “non-emergency”)	*System drivers of ED attendance:* • Inner circle of close relational ties did not greatly influence decision • Health professionals and wider health care system did influence considerably – some perceived them as expert and were influenced, others felt GP were ambiguous in their actions and risk adverse • Presence of a network member with authority and expertise often helped to reinforce the purpose of ED and push toward primary care
(Palmer, Jones et al. 2005) [91] UK Qualitative: Semi-structured telephone interviews *N* = 321 CASP = 10	Mean age: 36.6 ± 20.0 yr; Gender: males = 176, females = 145 Triage: Manchester Triage System (MTS)	• AED more appropriate than GP (38.3%) • GP would send me anyway (17.5%) • Referred by GP (22.4%) • Advised by others than GP (13.1%) • Quicker, wait too long for GP appointment (23.4%) • More convenient than GP (15.3%) • GP surgery closed/not available (30.5%) • No GP/GP more than 25 miles away (14.6%) • Already tried GP without good outcome (4.7%) • Other (1.6%)
(Pförringer, Pflüger et al. 2021) [103] Germany Qualitative, interview for open-ended questionnaire *N* = 235 CASP = 10	Age: < 30 yr = 88, 30–49 yr = 69, 50–67 yr = 49, > 67 yr = 29; Gender: males = 125, females = 110 Triage: Guidelines of the German Society of Traumatology	Descriptive statistics were used to gain quantitative statements: • Immediate help (45.9%) • Fast treatment by a specialist (35.4%) • Broad diagnostic tools (22.8%) • High quality treatment (17.9%) • Family doctor closed (12.6%) • Other (17.9%) • Fast admission to hospital (9.3%) • Attestation (5.7%) • Family doctor on holiday (4.4%) • Blood analysis (4.4%) • Free medication (3.3%) • Thorough consultation (2.8%) • Shorter waiting time (2.4%) Replacement of family doctor unknown (1.2%)
(Shaw, Howard et al. 2013) [89] USA Qualitative: Semi-structured Interviews *N* = 30 CASP = 10	Mean age: 40 yr (21–63 yr); Gender: males = 18, females = 12 Triage: Emergency Severity Index (ESI)	• No knowledge of other primary care options • Being instructed by a medical professional • Facing access barriers to their regular source of care • Perceiving racial issues with a primary care option • Defining their health care need as an emergency that required ED services • Facing transportation barriers to other primary care options
(van der Linden, Lindeboom et al. 2014) [90] Netherlands Observational: Structured interview by nurse *N* = 3028 CASP = 10	*Self-Referred:* Mean age: 32.3 ± 18.6 yr; Gender: males = 1636, females = 1392 Triage: Not specified (5-level triage system; included patients with levels 1–3 “life-threatening, very urgent, or urgent” and levels 4–5 “standard or non-urgent”)	Among the self-referred patients, 1751 answered the question (58%): • Accessibility and convenience • Perceived medical necessity • Not thought about going to the GP • Not having a regular GP • Familiarity • Dissatisfaction with GP • Referral by non-professionals • Language barriers
(Agarwal, Banerjee et al. 2012) [92] UK Qualitative: Semi-structured Interviews *N* = 23 CASP = 9	Age < 50 yr: Gender: males = 4, females = 6; Age 50–69 yr: Gender: males = 4, females = 2; Age 70 + : Gender: males = 4, females = 3 Triage: Not specified (initial assessment by an experienced consultant in the ED identified patients suitable to be cared for in an alternative service including primary care)	• Anxiety about their health and the reassurance arising from familiarity with knowledge of the emergency service • Issues surrounding access to general practice (e.g., no appointments, too long to wait) • Perceptions of the efficacy of the service (e.g., more thorough investigation) • Lack of alternative approaches to care
(Benger and Jones 2008) [85] UK Qualitative, semi structured questionnaire *N* = 200 CASP = 9	Mean age: 58 yr (16–91 yr); Gender: males = 96, females = 104 104 patients (52%) Triage: Not specified (authors excluded “triage category 1)”	Top five reasons why patients choose to attend ED (*N* = 57): • Perceived severity or urgency of their condition (51%) • Previous experience (12%) • Ease and convenience (7%) • Housebound (7%) • Primary care services are not available out of hours (7%)
(Read, Varughese et al. 2014) [105] Quatar Qualitative: Semi-structured interviews *N* = 100 CASP = 7	Mean age: 33 yr; Gender: females = 100 Triage: Not specified (non-urgent ED females classified with low-acuity conditions, excluding minor trauma and lacerations)	• Directed by employer to attend ED (40%) • Advised to come by family (35%) • Faster care and accessibility (98%)

*AED* Accident and Emergency Room, *ED* Emergency Department, *ER* Emergency Room, *GP* General Practitioner

**Table 3:** Study and sample characteristics, NIH quality appraisal score, and key themes/identified reasons for emergency department use among *quantitative* studies

**Study Characteristics** **Data Collection Method** **NIH Quality Appraisal Score**	**Sample Characteristics** **Formal Triage Method**	**Key Themes/Issues Identified for Reasons for Use**
(Afilalo, Marinovich et al. 2004) [63] Canada Observational: Secondary analysis of a prospective cross-sectional study *N* = 454 NIH = 7	Mean age: 43.3 ± 18.1 yr; Gender: males = 224, females = 230 Triage: Canadian Triage and Acuity Scale (CTAS)	• Accessibility (30.1%) • Perception of ED-specific need (22.1%) • Referral/follow up to the ED (20.2%) • Familiarity with the ED (11.1%) • Trust of the ED (7.4%) • No specific reason (7.1%)
(Amiel, Williams et al. 2014) [64] UK Survey Questionnaire *N* = 649 NIH = 7	Mean age: 35 yr (18–84 yr); Gender: males = 266, females = 383 Triage: Not specified (nurse streams patients into one of four categories: “minor illness,” “minor injury,” “emergency for transfer,” or “see and treat”)	• Quicker than a GP appointment (28%) • Nearest place to home or work (23%) • Best place for my particular problem (10%) • Recommended by friends, family or colleague (10%) • Thought there would be a shorter wait (8%) • More confidence in advice than given by own GP (7%) • Did not think about going anywhere else (6%) • Did not have a GP to go elsewhere (3%) • Wanted a second opinion (2%) • Other (3%)
(Cheek, Allen et al. 2016) [56] Australia Cross-sectional Survey *N* = 138 NIH = 7	Mean age: 47 ± 21.1 yr (18–87 yr); Gender: males = 63, females = 75 Triage: Australian Institute of Health and Welfare (AIHW)	Questions listed on the survey verbatim: • I am able to see the doctor and have any tests or x-rays all in the same place at the ED (71.7%) • My GP surgery was closed (57.2%) • I am not happy with the time I have to wait to acquire an appointment with a GP (34.8%) • The ED is closer to home or work than the GP surgery (34.8%) • I feel the medical treatment is better at the ED (32.6%) • I thought the GP would send me to the ED anyway (31.2%) • I have to wait too long to see the GP (29.7%) • I do not see the same GP when I attend my GP practice (29.0%) • My GP referred me to the Ed (16.7%) • I find it difficult to understand my GP (15.9%) • My family has traditionally used the Ed for our health care (15.9%) • I did not think my GP had the required equipment (15.2%) • I prefer the hospital environment to the GP surgery (14.5%) • I do not like making appointments and prefer the ED as I can attend when I want (13.0%) • I wanted to see a doctor I do not know (13.0%) • I wanted a second opinion (10.9%) • I am on holiday away from usual GP (2.9%) • I did not want GP to know about this particular problem (1.4%) • I preferred to see a female doctor and thought I could at the ED (0%)
(Coelho Rodrigues Dixe, Passadouro et al. 2018) [66] Portugal Cross-sectional survey administered via structured interview *N* = 357 NIH = 7	Mean age: 54.51 ± 20.9 yr (18 to 92 yr); Gender: males = 144, females = 213 Triage: Manchester Triage System (MTS)	• Disease justified ED use (91.7%) • Can undergo all medical examinations on same day (65.6%) • Wanted to be examined by specialist (53.9%) • Difficult to schedule an appointment at healthcare center (44.3%) • Quicker to be examined at hospital (38.1%) • Matter of habit (26.75%) • Unsatisfied with healthcare center in similar situations (26.6%) • Worsening of chronic disease during follow-up in outpatient visit (21.0%) • Healthcare center closed, did not know where to go (20.7%) • Doctor was not at the healthcare center, no alternative (16.4%) • No vacancy at healthcare center, I had no alternative (15.7%) • Visit hours at healthcare center weren’t compatible with work/school (15.4%) • Closer to the hospital (15.4%) • Don’t have family doctor (14.7%) • Hoping to be hospitalized (5.4%) • Have a private doctor, don’t usually use healthcare center (9.6%)
(Coleman, Irons et al. 2001) [26] UK Cross-Sectional Survey *N* = 255 NIH = 7	Age: < 35 yr = 145, > 35 yr = 110; Gender: males = 136, females = 119 Triage: Not specified (five-colour system (black, red, blue, green, yellow) with green meaning a new illness or injury that is non-urgent, yellow meaning a long-standing issue)	• Perceptions of seriousness (76%) • Positive experiences at ED (70%) • Seeking a specific service (68%) • Awareness of other services (62%) • Processes and patient’s time (56%) • Advised to come by others (43%) • Availability of other services (38%) • Seeking assurance (38%) • Convenience of access (24%) • Patient preference (11%)
(Ghazali, Richard et al. 2019) [72] France Cross-sectional survey *N* = 598 NIH = 7	Median age: 38 yr (IQR 27–50); Gender: males = 475, females = 123 Triage: French Emergency Nurses Classification in Hospital Scale, Classification Infirmière des Malades aux Urgences (CIMU)	• Expectation of getting hospital-based care, including access to further testing or hospitalization (*N* = 171) • Personal convenience (geographical proximity, opening hours) (N = 147) • Not having to pay for service (*N* = 20) Motivations: • Workplace accident (2.8%) • Suggested by peers (0.5%)/professional (9.7%) • Second opinion (3.6%) • Intense pain (4.5%) • Additional testing (26.3%) • Appointment hours (1.3%)/After business hours (5.2%) • Hospitalization (2.3%) • Unavailable primary care provider (19.2%) • Lack of upfront payment (3.7%) • Geographic proximity (17.7%) • Already taken care of in this hospital (3.2%)
(Han, Ospina et al. 2007) [31] Canada Questionnaire by either interview or self-administered, open-ended questions *N* = 894 (*N* = 421, 47% of CTAS 4–5) NIH = 7	Mean age: 44.1 ± 19.7; Gender: males = 438, females = 456 Triage: Canadian Triage and Acuity Scale (CTAS)	• Perceived severity of their health problems (*N* = 230) • Quality of care in the ED (*N* = 185) • Physician availability (*N* = 137) • Professional referral (*N* = 100) • Perceived rapidity of care in the ED (*N* = 80) • Felt it was only option (*N* = 76)/No physician available (N = 58) • ED was convenient (*N* = 71)
(Hodgins and Wuest 2007) [70] Canada Structured interviews *N* = 1612 NIH = 7	Mean age: 43.0 yr (16–93 yr); Gender: males = 629, females = 983 Triage: Not specified (“non-urgent” determined by a health professional)	16 total items; only 7 reported on by authors (no % provided) • Severity of symptoms (e.g., not willing to wait to see GP for pain) • Concern it will get worse • No other option • No availability of GP • Convenience of service • Needed service only available at ED • Tests only available at ED • Advised to come from family/friends
(Jalili, Shirani et al. 2013) [32] Iran Cross-sectional survey administered via structured interview *N* = 1923 (non-urgent = 400) NIH = 7	Age: 15–49 yr = 1571, > 50 yr = 727 (non-urgent: 15–49 yr = 334, > 50 yr = 66); Gender: males = 1196, females = 727 (non-urgent: males = 242, females = 158) Triage: Canadian Triage and Acuity Scale (CTAS)	• Obtaining rapid care (77%) • Proximity (52.8%) • Low cost (20.8%) • Unavailability of clinic area (19.8%) • Better care (11.3%) • Perception of urgent problems/urgency of the problem (10.8%) • Having medical records in this hospital (10.3%) • Being referred by a clinic or office (7.3%) • Being an employee of this hospital (7.3%) • Dissatisfaction with clinic or office (4.5%) • Being brought by EMS ambulance (0.5%) • No reasons mentioned (0.5%) • Miscellaneous (0.5%)
(Lee, Lau et al. 2000) [75] Hong Kong Telephone interviews, using questionnaires *N* = 1374 NIH = 7	Age: 0–19 yr = 561, 20–64 yr = 728, 65 + = 85; Gender: males = 735, females = 639 Triage: Not specified (blind retrospective review of patient charts conducted by an independent panel of emergency physicians; patients were divided into two categories (i.e., accident and emergency cases or GP-type cases)	• Could not afford GP (61.2%) • Proximity (21.2%) • Better quality service at ED (13.4%) • Efficient diagnosis (2.9%) • Symptoms getting worse (0.1%)
(Lobachova, Brown et al. 2014) [35] USA Cross-Sectional Survey *N* = 1062 NIH = 7	Mean age: 43.0 ± 22.0 yr; Gender: males = 552, females = 510 Triage: Not specified	• I believed that my problem was serious (61%) • My care provider told me to come (35%) • I thought it was an emergency (26%) • My illness occurred after hours (21%) • It was suggested by family/friend (13%) • I have no primary care provider (8%) • I thought it was unnecessary to contact my regular provider (8%) • The ED is convenient (8%) • My primary care provider is not from here (7%) • I could not get an appointment with MD (6%) • I spoke to a specialist (5%) • I did not know where else to go (3%) • I don’t know (0.5%) • I have no insurance (1%) • Other (16%) • Unspecified (16%)
(Marco, Weiner et al. 2012) [36] USA Cross-Sectional Structured Survey via Interview *N* = 292 NIH = 7	Age: 18–39 yr = 140, 40–64 yr = 100, 65 + = 49; Gender: males = 136, females = 156 Triage: Not specified	• Convenience/location (41%) • No GP (37%) • Institutional preference (23%) • Emergency medical condition (19%) • Issues with primary care (e.g., lack of available appointments, couldn’t get through, long wait, no on-call) (17%) • Physician referral (14%) • Primary care institutional affiliation (12%) • Don’t know, didn’t think about it, no reason (6%) • Other (7%)
(Masso, Bezzina et al. 2007) [37] Australia Cross-Sectional Survey *N* = 397 NIH = 7	Mean age: 38 yr (0–96 yr); Gender: males = 222, females = 175 Triage: Australasian Triage Scale (ATS)	• My health problem required immediate attention (67.3%) • I am able to see the doctor and have any tests or X-rays all done at the same place (51.3%) • My health problem was too serious or complex to see a GP (38.2%) • I feel the medical treatment is better at the ED (15.4%) • I am not happy with the time I have to wait to get to an appointment with a GP (12.6%) • It is easier for me to go to the ED” (8.4%) • I am not able to get in as a patient at GP surgery as the books are closed (7.6%) • I wanted a second opinion (5.7%) • I do not like making appointments (4.2%) • I usually prefer to talk a doctor a don’t know about my health problems (3.4%) • I did not want my GP to know about this health problem (1.6%)
(Miyazawa, Maeno et al. 2019) [73] Japan Cross-sectional survey *N* = 231 (Reported on Non-urgent ED subset = 84) NIH = 7	Mean age: 43.5 ± 18.5 yr; Gender: males = 51, females = 33 Triage: Japan Triage and Acuity Scale (JTAS)	Inappropriate use group (*N* = 84) • Desired to be cured quickly (92.5%) • Wanted a doctor’s opinion (90.6%) • Wanted to know whether the condition was serious (83.9%) • Condition was not improving (80.6%) • Wanted a prescription (76.7%) • Wanted a laboratory test done (65.1%) • Desire for treatment by a specialist (59.3%) • Recommended by others (45.8%) • Over-the-counter medicine was not working (35.6%) • Wanted to know if they could attend work, school, events (24.1%) • Wanted an intravenous drip (20.7%) • Inability to take time off from school or work during the day (38.7% of inappropriate group)
(Penson, Coleman et al. 2012) [43] UK Observational: Survey *N* = 261 NIH = 7	Age: 14–34 yr = 108, 35–55 yr = 77, 55 + yr = 77; Gender: males = 140, females = 121 Triage: Not specified (“minor” injury were fined by a list of explicit criteria	Ranges reflect the sub-themes of reasons within each overall category endorsed by patients: • Availability of other services (i.e., no GP or no availability) (6–69%) • Awareness of other services (i.e., not sure where to go, unsure of other services, when open) (16–46%) • Patient preferences (i.e., not wanting to see their GP, can’t always see the same one, not wanting to bother them) (6–15%) • Positive experiences of ED (i.e., confident, happy) (60–74%) • Processes and patient’s time (i.e., GP would refer to ED anyway, seen quicker, do not have to wait for appointment) (17–48%) • Convenience of access (i.e., location, ease) (18–29%) • Perceptions of seriousness (21–98%) • Reassurance (91%) • Second opinion (25%) • Directed by others (36–78%) • Seeking particular services (4–84%)
(Schumacher, Hall et al. 2013) [45] USA Structured interviews based on a survey *N* = 492 NIH = 7	Mean age: 41 ± 17 yr; Gender: males = 221, females = 271 Triage: Emergency Severity Index (ESI)	• Right place to go (92%) • Emergency (89%) • Worried (93%) • Too much pain (73%) • Too sick or injured (52%) • Do not like usual (13%) • Medical records are at ED (41%) • Better care at the ED (61%) • Always get care in ED (47%) • Like environment of the ED (25%) • No insurance (21%) • Financial (22%) • MD-refused insurance (3%) • One stop (63%) • No appointment necessary (45%) • Closest or easiest place (54%) • No place to go (55%) • Only place open (26%) • Language (33%) • Family or friends (32%)
(Ward, Huddy et al. 1996) [69] UK Cross-sectional survey (single question) *N* = 970 NIH = 7	Age range: 21–30 yr (344/965 patients with complete data); Gender: not reported Triage: Not specified	Question answered by 339 patients: • Problem not appropriate for GP (27.1%) • Not convenient to see GP (22.4%) • Advised by health professional 39 (11.5%) • Second opinion (9.7%) • Did not try to see GP (9.7%) • Appointment not available with GP (7.4%) • Unable to contact GP (6.2%) • Dissatisfied with GP (4.4%) • Other (1.5%)
(Watson, Ferguson et al. 2015) [53] UK Cross-sectional survey *N* = 81 NIH = 7	Mean age: 42.2 ± 17.9 yr; Gender: males = 36, females = 43; missing = 2 Triage: Not specified (non-urgent patients determined to have a “common or self-limiting or uncomplicated conditions which may be diagnosed and managed without medical intervention”)	Major categories (range reported by subcategories of reasons) • Convenient location (1.2%-51.9%) • Knowing, feeling comfortable, or trusting the staff (1.2%-34.6%) • Condition too serious to go to GP or chemist (27.2%-30.9%) • Previously attended GP or chemist but condition not improved (3.7%-16.0%) • Have to wait longer for a GP appointment (37.0%) • Prefer not to go to GP or chemist (3.7%-4.9%) • Cost of treatment (1.2%)
(Afilalo, Guttman et al. 1995) [16] Canada Cross-sectional survey administered via structured interview *N* = 849 (*N* = 186 for Category II and III interviews) NIH = 6	Total sample: Age: < 65 = 72.7%; Gender: males = 418, females = 431 Triage: Not specified (three-level list of explicit criteria)	• Other clinic is closed (25.0%) • Perception of serious illness (20.7%) • Familiarity or trust in the ED (12.1%) • Proximity (10.7%) • Unaware of services available elsewhere (8.6%) • Dissatisfied with other out-patient facilities (8.6%)
(Al-Otmy, Abduljabbar et al. 2020) [17] Saudi Arabia Cross-sectional survey administered via structured interview *N* = 400 (*N* = 314 non-urgent) NIH = 6	Total Sample: Mean age: 50.3 ± 19.7 yr (14–98 yr); Gender: males = 181, females = 219 Triage: Canadian Triage and Acuity Scale (CTAS)	For those triaged as non-urgent (*N* = 314) • Participant felt their condition was urgent (41.1%) • Easier accessibility (26.1%) • Limited resources and services in the primary healthcare centre (19.4%) • Difficulty getting an appointment (11.8%) • Referred from primary healthcare centre to ED (3.5%)
(Alyasin and Douglas 2014) [18] Australia Cross sectional survey *N* = 350 NIH = 6	Mean age: 32.1 ± 12.2 yr (18 to 80 yr); Gender: males = 202, females = 148 Triage: Canadian Triage and Acuity Scale (CTAS)	• Do not have a regular healthcare provider (63.4%) • Can receive care on the same day without an appointment (62.6%) • Convenience and access to medical care 24/7 (62.6%) • ED gives better care than other health services in the area (44.6%) • Can access investigation such as blood tests/x-rays (37.4%) Urgency of problem (22.3%)
(Atenstaedt, Gregory et al. 2015) [55] UK Cross-Sectional Survey *N* = 806 NIH = 6	Age: 0–15 yr = 12%, 16–29 yr = 27%, 30–69 yr = 57%, 75 + yr = 4%; Gender: males = 459, females = 347 Triage: Manchester Triage System (MTS)	• Thought might need radiograph (46%) • Did not think GP could help (29%) • GP was not available (19%) • Could be seen quicker at ED (11%) • Thought might need to go to hospital (10%) • Wanted to see specialist (9%) • Thought might need stitches (6%) • ED nearer than other service (6%) • Was not aware of other services (3%) • Does not have GP (3%) • Did not want to bother GP (3%) • Wanted a second opinion (3%) • Thought might need tetanus shot (3%) • ED is easier to get to than other service (2%) • Dentist was not available (1%) • Thought might need blood test (1%)
(Baker, Stevens et al. 1995) [20] USA Cross-sectional survey *N* = 1190 NIH = 6	Mean age: 37 yr ± 14.0 yr; Gender: males = 524, females = 666 Triage: Not specified (four-level triage system based on a list of explicit criteria)	• Among 58% sample who attempted to see their GP, they failed due to cost (43%), lack of insurance (36%), and inability to obtain an appointment rapidly (19%) • Among 38% who did see their GP in the preceding week, 68% were referred to ED • Among all patients, 89% said that they needed to be seen immediately
(Burchard, Oikonomoulas et al. 2019) [25] Germany Cross-sectional survey *N* = 499 NIH = 6	Median Age: 32 yr (IQR 50–22); Gender: males = 300, females = 199 Triage: Manchester Triage System (MTS)	• Deemed their medical condition something that needed urgent or emergency diagnosis and treatment (63.1%) • A GP would be unable to treat their medical problem (74%) • Expected a hospital admission or in-patient treatment was necessary (2.4%) • Factors guiding decision (ED over GP): • Technical equipment (3.5%) • No GP (1.4%) • 24/7 Access (4.3%) • Negative experience (0.4%) • Waiting experience (10.3%) • I do not like to answer this question (80.1%)
(Barbadoro, Di Tondo et al. 2015) [21] Italy Cross sectional survey *N* = 61 NIH = 6	Age: 18–65 yr = 52, ≥ 65 = 9; Gender: males = 33, females = 28 Triage: Not specified (“non-urgent” patients defined as having no active symptoms or were recent and minor, without any feeling of emergency and he/she desires a check-up, a prescription refill or a return-to work release)	Of the non-urgent participants (*N* = 61), the following were present motivations for accessing ED: • Urgency perceived by patient (*N* = 23) • Recent traumatic injury (*N* = 14) • Difficulty contacting GP (*N* = 9) • Greater confidence in the hospital (*N* = 14) • Previous medical therapy without benefit (*N* = 10) • Too long to book exams (*N* = 20) • ED has more tools to solve clinical problems (*N* = 21) • Easy accessibility of ED (*N* = 5)
(Dawoud, Ahmad et al. 2015) [57] Saudi Arabia Cross sectional study, Interviewed with structured questionnaire *N* = 300 NIH = 6	Age: ≤ 15 yr = 80, 16–31 yr = 105, 32–60 yr = 93, > 60 yr = 22; Gender: males = 152, females = 148 Triage: Canadian Triage and Acuity Scale (CTAS)	Reasons why patients went to ER instead of primary healthcare center: • Limited working hours (60.8%) • Limited services and resources (60.4%) • Mistrust of health centers (24.6%) • Lack of experience among the medical staff (10.1%) • Lack of knowledge of the health centers (7.1%) • Dissatisfaction with the treatment provided (7.1%) • Lack of effective diagnosis (6.3%) Reason why patients went to ER despite having health insurance: • Closest governmental hospital (69.8%) • Other hospital does not receive some cases (44.4%) • Congestion in other hospitals (14.3%) • Insurance requirements have not yet been completed (12.7%) • Trust the governments treatment more (4.8%)
(de Valk, Taal et al. 2014) [27] Netherlands Cross-Sectional Survey *N* = 436 NIH = 6	Age: 18–35 yr = 54, 35–65 yr, 65 + = 7; Gender: males = 251, females = 185 Triage: Not specified	• Belief that ED could provide care that the GP could not (28%) • Specialist that patient sees already at that hospital (17%) • There was not a GP nearby (16%) • Could get help earlier at ED (15%) • ED was located nearby (11%) • Did not have a GP (11%) • Could not contact the GP (7%) • Unsure where to locate a GP (5%) • Previous negative experience with GP (4%) • No trust in GP (3%) • Advised by others to go (3%) • Belief the complaint was urgent (2%)
(Diserens, Egli et al. 2015) [76] Switzerland Observational: Survey *N* = 516 (2000) *N* = 581 (2013) NIH = 6	Sample from 2000: Mean age: 46.4 ± 22.0 yr; Gender: males = 294, females = 222 Sample from 2013 Mean age: 44.5 ± 20.0 yr; Gender: males = 314, females = 267 Triage: Swiss Emergency Triage Scale (SETS)	Reasons for Self-Referral to ED (2000 vs. 2013) • Unawareness of alternatives for emergencies (12.5% vs. 5.4%) • Excellence of the institution and access to specialists (9.8% vs. 3.8%) • Usual place of consultation (6.7% vs. 4.1%) • Easy access (3.4% vs. 5.2%) • Dissatisfaction with treatment or appointment with GP (0.7% vs. 1.7%) • Convenience of unscheduled appointment (0.5% vs. 1.7%) • Paramedics choice (0.5% vs. 1.7%) • Other (0.7% vs. 1.3%)
(Field and Lantz 2006) [29] Canada Cross-section survey *N* = 235 NIH = 6	Age: not reported; Gender: not reported Triage: Canadian Triage and Acuity Scale (CTAS)	• Access to a specific service (49%) • Obtain rapid treatment for a perceived urgent problem (43%) • Limited access to family physician (23%) • Referred to the ED (20%) • Did not have a family physician (3%)
(Gentile, Vignally et al. 2010) [71] France Cross-sectional survey *N* = 85 NIH = 6	Mean age: 36.3 ± 11.7 yr (18–70 yr); Gender: males = 50, females = 35 Triage: Not specified (patients deemed “non-urgent” by triage nurse)	• Were unable to contact GP (33%) or trouble accessing their usual source of care (22.3%) • Referrals: self (76%), GP (17.6%), for medico-legal reasons by employer/police (5.9%) • Attending due to the pain (65.8%) • Need for diagnostic investigations (37.6%) • Needing consultation for traumatological problems
(Gill and Riley 1996) [30] USA Cross-Sectional: Structured interview *N* = 268 NIH = 6	Age: 18–39 yr = 138, 40–64 yr = 54, 65 + yr = 5; Gender: males = 132, females = 135, unknown = 1 Triage: Not specified (non-urgent patients defined as those who “may safely wait several hours or more for evaluation”)	Reasons for attending ED (perceived urgency: urgent vs. non-urgent): • Emergency department closer (33 vs. 39%) • Emergency department faster (19 vs. 25%) • No regular source of care (19% vs. 16%) • Likes emergency department service (16% vs. 18%) • Regular source of care not accessible (20% vs. 8%) • Urgent problem (16% vs. 14%) • Referred (11% vs. 16%) • More convenient (11% vs. 12%) • Financial (7% vs. 8%) • Better medical care (6% vs. 6%)
(Idil, Kilic et al. 2018) [81] Turkey Cross-sectional survey *N* = 624 NIH = 6	Mean age: 38.4 ± 14.4 yr; Gender: males = 326, females = 298 Triage: Not specified (three-level colour system with green indicating lowest urgency; patients do not require urgent interventions and could be treated outside the ED in polyclinics or by their family physicians)	• Able to get examined more quickly (36.4%) • Not being able to book early appointments with alternative health units (30.9%) • No given reason for preference to the ED (20.2%) • ED is physically closer than the family physician (12.8%) • Visited ED for complaints when they were at hospital for a different reason (12.3%) • Other reasons (get medications prescribed, get incapacity report, or seek medical counselling services, etc.) (8.0%)
(Jiang, Ye et al. 2020) [33] China Cross-sectional survey *N* = 545 NIH = 6	Age: > 18 = 152, 19–44 = 217, 45–64 = 123, > 65 = 53; Gender: males = 271, females = 274 Triage: Modified Emergency Severity Index (ESI)	• Perceived severity of illness and urgent treatment needed (68.6%) – illness is severe, advised by family/friends, need reassurance for their condition • Poor access of alternative services (26.4%) – can’t get appointments, can’t get specific services elsewhere, alternatives not opened at this hour • Referral by medical staff (24.6%) • Convenience and advantages of ED services (21.5%) – easier to get appointment, evaluated/treated quickly, quality of care is superior, staff qualifications • Unsure where else to go (4.6%) • Regard ED as a regular medical resource (4.4%) • Other reasons (0.4%)
(McGuigan and Watson 2010) [38] UK Cross-Sectional: Semi-structured telephone interviews *N* = 196 NIH = 6	Age: Not reported; Gender: Not reported Triage: Not specified	• Perceived appropriateness of condition (48%) • After taking advice from others (mostly family) (35%) • Anticipation of referral by GP (3%) • Accessibility of ED (6%) • Unavailability of GP (5%) • Other (1%)
(Moll van Charante, ter Riet et al. 2008) [79] Netherlands Postal questionnaires *N* = 224 NIH = 6	Median age: 33 yr (IQR 30); Gender: males = 175, females = 49 Triage: Not specified	• Additional investigations were necessary (36%) • ED physician is best qualified for the problem (30%) • ED is more accessible than the GP (16%) • Related to a recent hospital contact or procedure (5%) • Did not want to disturb the GP or no GP available (4%) • Other (5%) • No response (4%)
(Nelson 2011) [80] Scotland UK Telephone interviews using structured questionnaire *N* = 27 NIH = 6	Age: 16–40 yr = 20, 40 + = 7; Gender: males = 13, females = 14 Triage: Not specified	• Need for x-rays (37%) • Referred by their GP (15%) • Advised by the health centre receptionist to attend the ED (7%) • Unable to obtain a GP appointment (4%)
(Norredam, Mygind et al. 2007) [67] Denmark Cross-sectional survey *N* = 3426 NIH = 6	Mean age: 0–14 yr = 617, 15–24 yr = 624, 25–44 yr = 1343, 45 + = 781; Gender: males = 1925, females = 1501 Triage: Not specified	• The ED is most relevant to my need (63%) • I was referred by a primary caregiver (24%) • I could not get in contact with a GP (13%)
(Northington, Brice et al. 2005) [39] USA Cross-sectional survey *N* = 279 V6	Mean age: 37.4 ± 14.9 yr; Gender: males = 154, females = 125 Triage: Emergency Severity Index (ESI)	• Better care (76.1%) • Urgency (73.6%) • Immediacy (68.6%) • Payment flexibility (41.9%) • Expediency (39.7%)
(Oetjen, Oetjen et al. 2010) [41] USA Cross-Sectional: Survey questionnaire *N* = 438 NIH = 6	Age: 2–18 yr = 127, 19–50 yr = 197, 50–80 yr = 114; Gender: males = 29%, females = 70% Triage: Not specified (non-urgent defined as “those cases in which the patient does not require immediate care or attention within a few hours”)	• Patient believed condition was serious (72%) • Primary care physician referred them (57%) • After-hours (9%) • Insurance (8%) • ED was more convenient: quality (10%) • ED was more convenient: location (14%) • ED was more convenient: staff (51%) • Friends recommended coming (9%)
(Oktay, Cete et al. 2003) [42] Turkey Cross-sectional survey *N* = 1155 NIH = 6	Mean age: 44.9 ± 18.1 yr; Gender: males = 503, females = 652 Triage: Canadian Triage and Acuity Scale (CTAS)	• Proximity to ED (19.8%) • Satisfaction with care (12.5%) • Pain and worsening of symptoms (11.5%) • Clinic care unavailable (11.3%) • Quick care and laboratory results (8.5%) • Always get care in this hospital (7.6%) • Perception of serious illness (6.4%) • Told to go to ED by relatives or others (4.7%) • Trust out ED care (2.8%) • Thought symptoms would become intensified (2.6%) • Told to come to our ED for follow up (2.4%) • Relatives work in our ED (2.1%) • Miscellaneous (7.8%)
(O’Loughlin M 2019) [40] Australia Cross-sectional survey *N* = 1000 NIH = 6	Mean age: 48.6 ± 19.0 yr; Gender: males = 493, females = 507 Triage: Not specified (non-urgent patients were those with “potentially avoidable general practitioner (PAGP)-type presentations”)	• No choice/urgent problem (35.5%) • Best place for problem (25.0%) • Services in one location (11.6%) • Open 24 h (4.6%) • Quicker than a general practice (3.2%) • Need admission (2.6%)
(Ragin, Hwang et al. 2005) [68] USA Questionnaires and interviews *N* = 1536 NIH = 6	Mean age: 45.9 ± 19.3 yr; Gender: males = 685, females = 851 Triage: Not specified	• Medical necessity – perceived ED was the place to be (95.0%) • Convenience (86.5%) • Preference of ED over alternate services (88.7%) • Affordability (25.2%) • Limitations of insurance (14.9%)
(Redstone, Vancura et al. 2008) [59] USA Cross-sectional survey *N* = 240 NIH = 6	Mean age: 45 yr; Gender: males = 76, females = 164 Triage: Emergency Severity Index (ESI)	• Could not wait 1–2 days (93%) • ED more convenient (62%) • Need a test not available at GP (51%) • Problem too complex for GP (45%) • Advised to go to ED (49%) • Perceived need of hospital admittance (24%)
(Selasawati, Naing et al. 2007) [46] Maylasia Cross-sectional survey *N* = 170 (case) *N* = 170 (control) NIH = 6	Case (ED Patients; *N* = 170): Mean age: 36.7 ± 13.6 yr; Gender: males = 97, females = 73 Control (Outpatients; *N* = 170): Mean age: 40.2 ± 14.6 yr; Gender: males = 46, females = 124 Triage: Triage guideline of Hospital Kuala Lumpur (HKL) and Hospital University Kebangsaan Malaysia (HUKM), American College of Emergency Physician (ACEP) and ED criteria of Davis Medical Centre	• Due to severity of illness (85%) • Can’t go to OPD during office hours (42%) • ED near house (27%) • Better treatment in ED (26%) • Staff or family member (17%) • No other place to go (15%) • Financial problem (8.8%)
(Shah, Shah et al. 1996) [47] Kuwait Cross-Sectional Survey *N* = 1986 (*N* = 1212 non-urgent, self-referred only) NIH = 6	(Non-urgent, self-referred only; *N* = 1212): Age: < 25 yr = 266, 25–34 yr = 392, 35–49 yr = 349, 50 + = 205; Gender: males = 691, females = 521 Triage: Not specified (4-level triage system from emergency level 1 to non-urgent level 4)	Preference • ED better or clinic worse/medicine not available (27.8%) Accessibility/availability • Accessibility/availability of ED (59.8%) • Hospital staff (14.0%) • Clinic closed/not available/do not know clinic schedule (7.5%) • ED close by or convenient (13.2%) • Regular patient (12.1%) • Refused by primary care physician (2.0%) Perceived Urgency • Perceived condition to be urgent (10.7%) Other (1.6%)
(Siminski, Cragg et al. 2005) [48] Australia Cross-sectional Survey *N* = 400 NIH = 6	Mean age: not reported; Gender: not reported Triage: Australian Triage Scale (ATS)	• Problem too urgent (80%) • See doctor and testing done in same place (74%) • Problem too serious/complex (53%) • Medical treatment better at ED (34%) • Not happy with GP waiting time (24%) • Easier to get to the ED (21%) • Not able to see GP as books are closed (16%) • Second opinion (14%) • Do not like making appointments (12%) • No charge for X-rays or medicine (10%) • No charge to see a doctor (9%) • Traditional use by family (9%) • Prefer doctor I don’t know (6%) • Prefer ED environment (5%) • Did not want the GP to know (2%) • Female doctor (2%) • Doctor/interpreter with native language (2%) • Aboriginal health staff (2%)
(Steele, Anstett et al. 2008) [49] Canada Cross-sectional survey *N* = 137 NIH = 6	Mean age: not specified; Gender: not specified Triage: Canadian Triage and Acuity Scale (CTAS)	• Needed treatment as soon as possible (38.7%) • Needed a specific service offered in the ED (32.8%) • Walk-in clinic was closed (24.8%) • Family physician’s office was closed (21.9%) • Could not wait for appointment with family physician (16.8%) • Did not have a family physician (4.4%)
(Thornton, Fogarty et al. 2014) [50] New Zealand Cross-sectional survey *N* = 421 NIH = 6	Mean age: 37.6 ± 24.6 yr; Gender: males = 203, females = 218 Triage: Australasian Triage Scale (ATS)	• Among those who contacted their GP (25%), they were advised to go to ED (73%) • GP was closed (29%) • Felt sick enough to require ED care (32%)
(Unwin, Kinsman et al. 2016) [51] Australia Cross-sectional survey *N* = 477 NIH = 6	Age: < 25 yr = 217, > 25 yr = 260; Gender: males = 224, females = 253 Triage: Australian Triage Score (ATS)	• It was clearly an emergency to me (37.1%) • Patient may need to have tests (such as x-rays and/or blood tests) (40.3%) • ED more available than GP or other health care service (28.7%) • GP not available (35.8%) • Patient was told to go to ED by a doctor or nurse (28.9%) • A health help line indicated the patient should attend (5.0%) • It was related to a recent hospital contact or procedure (5.7%) • Other services are too expensive (6.9%) • The patient uses the ED for all their health concerns (2.1%) • Did not know where else to go (9.2%) • Other (6.9%)
(Wang, Tchopev et al. 2015) [52] USA Cross-sectional survey *N* = 2711 NIH = 6	Female mean age (*N* = 1746): 26.7 ± 17.5 yr; Male mean age (*N* = 965): 19.9 ± 19.6 yr Triage: Not specified	Health care service delivery issues: • Access (11.0%) • Primary care provider unavailable (44.9%) Population behaviour issues • Dissatisfaction with primary care provider (0.6%) • Medication needs (0.2%) • Unaware of primary care provider (0.8%) • Usual place of care (0.3%) Unavoidable ED visits • Acute conditions (38.2%) • Referral by primary care provider (4.1%)
(Young, Wagner et al. 1996) [54] USA Cross-sectional survey *N* = 6187 NIH = 6	Median age: 31 yr, < 18 yr = 24%; Gender: males = 3046, females = 3141 Triage: Not specified (non-urgent patients determined to be those who came to ED but were 1) routed to an adjacent fast track unit, 2) rerouted to an urgent care clinic nearby, or 3) those refused care and were turned away after triage)	• Emergent or urgent condition (39%) • Told to go to ED by clinician (19%) • Too sick to go elsewhere (6%) • Get good care in the ED (11%) • Get diagnosis and/or treatment (11%) • Barriers to receiving care elsewhere (65%) • Clinic not open at night/not get off work (11%) • Nowhere else to go for care (11%) • Geographical reasons (8%) • Tried to get care elsewhere (4%) • Transportation problems (3%) • Clinic does not take walk-in patients (3%) • No money or insurance (8%) • Free or low-cost ED care (4%) • Insurance or work requirement (2%) • Insurance pays for ED care (1%)
(Baskin, Baker et al. 2015) [22] USA Cross-sectional survey *N* = 59 NIH = 5	Mean age: 43.5 ± 14.8 yr (18–91 yr); Gender: Not reported Triage: Not specified	Percentage of sample that agreed with the statement: • Sought treatment from a health care provider before accessing ED services (20%) • Too worried about problem (97%) • ED is the right place to go for problem (90%) • Medical emergency (85%) • Too sick/injured to go elsewhere (85%) • In too much pain (85%) • ES is closest/easiest place (81%) • No appointment necessary (76%) • Everything can be done at one place (49%) • No place other than ED (48%) • Regular care at this hospital (41%) • They have no insurance (39%) • Cannot afford other places (36%) • Their medical record is there (32%) • Family/friend told me to come (19%) • Like environment of the ED (10%) • ED is only place open (3%) • Other places don’t take my insurance (3%) • Better medical care here (3%) • Need prescriptions refilled (3%)
(Bahadori, Mousavi et al. 2019) [19] Iran Cross-sectional survey administered via structured interview *N* = 1217 NIH = 5	Age: < 49 yr = 777, > 49 yr = 440; Gender: males = 675, females = 542 Triage: Canadian Triage and Acuity Scale (CTAS)	• Proximity (8.5%) • Closure of other centres or offices (3.2%) • Being referred by a clinic or a physician’s office (8.4%) • Having medical records in this hospital (29.5%) • Perceived urgent problems/urgency of the problem (5%) • Receiving better-off quality care (3.4%) • Dissatisfaction with the clinic or physicians’ offices (2%) • Receiving prompt care (36.6%) • Seeking lower costs and cheaper care (36%) • Transported by EMS ambulances (0.3%) • Being an employee at hospital (patient or family member) (1.8%) • No reasons provided (1.4%) • Others (4.8%)
(Becker, Dell et al. 2012) [74] South Africa Cross-Sectional: Questionnaire by Masso et al. 2010 *N* = 277 NIH = 5	Mean age: 31.5 yr; Gender: males = 122, females = 155 Triage: South African Triage Score	The common self-reported reasons for attending the ED were: • the clinic medicine was not helping (27.5%) • a perception that the treatment at the hospital was superior to that at the clinic (23.7%) • lack of a primary health clinic service after-hours in a specific geographical location (22%) • too-long clinic waiting times (14%); (v) patients being referred to the EC (12.3%) • that patients could have ‘special tests’ at the hospital (11.9%)
(Bianco, Pileggi et al. 2003) [23] Italy Cross-sectional Survey *N* = 106 NIH = 5	Mean age: 50.6 yr (15–98 yr); Gender: males = 44, females = 62 Triage: Not specified (four-level system with a list of explicit criteria created a priori for this study)	• Most frequent reason stated for the visit was that they believed it was an emergency; more frequently indicated by patients judged to be presenting with non-urgent conditions (91%) compared with other patients (81.3%)
(Brasseur, Gilbert et al. 2021) [65] Belgium Cross-sectional survey *N* = 1326 NIH = 5	Mean age: 39.8 ± 24.55 yr; Gender: males = 970, females = 975 Triage: ELISA Scale	• Suitability: ED appropriate for current problem (51.3%) • Accessibility: Easily accessible (23.8%) • Reputation: Felt confident about being cared for in the ED/ Felt specialized care was needed or because patient was being followed by a specific service from this hospital (4.6%) • Because of the stress (4.2%) • Financial concerns (0.8%) • Others (15.3%)
(Brim 2008) [24] United States Cross-sectional survey *N* = 64 NIH = 5	Mean age: 36 yr (18 – 76 yr); Gender: males = 24, females = 40 Triage: Not specified (“non-urgent” patients defined as requiring minimal procedures, medications or treatments, having minimal to no alteration in vital signs, and can wait without compromise)	Open-ended question – any comments you would like to make about the reason you selected the ED for your care today? (*N* = 33): • Lack of providers open to publicly insured or uninsured participants (N = 9) • Long waiting times for appointments (*N* = 8) • Need for help (*N* = 6) • Sense of urgency for care (*N* = 8)
(Faulkner and Law 2015) [28] Australia Quantitative/Qualitative—Telephone interviews with open and closed-ended questions *N* = 58 NIH = 5	Age: 65–74 yr = 35, 75–89 yr = 20, 90 + = 3; Gender: males = 27, females = 31 Triage: Australian Institute for Health and Welfare (AIHW)	• Condition was serious and needed urgent attention (29.1%) • Only place open (17.1%) • GP sent me to ED (12.8%) • Was the weekend (10.3%) • Could not get into local GP (6.0%) • ED has more facilities (8.5%) • Other (16.2%)
(Graham, Kwok et al. 2009) [78] Hong Kong Cross-sectional survey administered via structured interview *N* = 249 NIH = 5	Mean age: 44 ± 18 yr; Gender: males = 126, females = 123 Triage: Hospital Authority of Hong Kong, Accident and Emergency Department Triage Guidelines	• Desire for more detailed investigations (56%) • Perception that more professional medical advice would be given in ED (35%) • Patient currently under continuing care at same hospital (19%) • Direct referral from other health care professional (11%) • Do not need to pay a fee (1.2%) Unaware of availability of general outpatient clinics (5.7%)
(Hunt, DeHart et al. 1996) [58] USA Cross-Sectional Survey *N* = 1547 NIH = 5	Mean age: Not Reported; Gender: Not Reported Triage: Not specified (patient severity determined by the physician after they had been assessed and treated)	Columbia Grand Strand Regional Medical Center (tourist community) – 6 most frequent reasons (*N* = 557): • I’m from out of town and just looked for the nearest emergency room. (23.0%) • Don’t have a doctor/clinic that regularly takes care of me. (21.7%) • Don’t have to make an appointment at the emergency room. (20.1%) • Better medical care here than other places. (15.7%) • My problem is bigger than my regular doctor/clinic could take care of. (14.6%) • My doctor/clinic told me to come to the emergency department when the office is closed. (12.0%) Pitt County Memorial Hospital (training program) – 6 most frequency reasons (*N* = 990): • Don’t have a doctor/clinic that regularly takes care of me. (15.6%) • Better medical care than places. (14.3%) • Don’t have to make an appointment at the emergency room. (12.7%) • My doctor/clinic told me to come to the emergency department when the office is closed. (11.0%) • My doctor couldn’t see me soon enough. (7.6%) • My problem is bigger than my regular/clinic could take care of. (7.1%)
(Laffoy, O'Herlihy et al. 1997) [34] Ireland Cross-Sectional: Structured interview questionnaires *N* = 557 NIH = 5	Age: 0–15 yr = 10, 15–44 yr = 367, 45–74 yr = 128, 75 + = 30; Gender: not reported Triage: Not specified	• Thought I needed immediate attention (35.4%) • Thought I needed an X-ray (18.2%) • Hospital is convenient (13.7%) • Thought GP would refer me anyway (7.6%) • I prefer hospital for this condition (7.1%) • I’m under hospital care already (5.6%) • Hospital cheaper than GP (0.8%) • GP told me to go to ED (0.3%) • Other (14.4%)
(Müller, Winterhalder et al. 2012) [77] Switzerland Cross-Sectional Survey *N* = 200 NIH = 5	Mean age: 35.5 yr (15–83 yr); Gender: males = 129, females = 71 Triage: Not specified	• Didn’t want to disturb GP (2.5%) • ED can help better (14.0%) • ED has better infrastructure (14%) • GP is too far away (9%) • I couldn’t reach the GP (15%) • I have no GP (10.5%) • Low confidence in GP (2.5%) • Other (12%)
(Rassin, Nasie et al. 2006) [83] Israel Cross-sectional survey *N* = 73 NIH = 5	Mean age: 39.4 yr (18–82 yr); Gender: males = 44, females = 29 Triage: Not specified	• Recommendation of a family member (68.6%) • Quality of ED greater than primary care (62.9%) • Geographical proximity to their home (47.2%) • Usually when they feel sick they go to the ED (43%)
(Walsh 1995) [61] UK Qualitative and Quantitative: Structured interviews *N* = 200 NIH = 5	Age range: 16–60 yr; Gender: males = 100, females = 100 Triage: Not specified (non-urgent patients defined by presentation to “minor injury” section of an ED)	• ED more appropriate or better than GP (20%) • GP would send me here anyway (17%) • Quicker/wait too long for GP appointment (17%) • Sent by GP after initially going to GP (14.5%) • Advised to go to ED by others than GP (13.5%) • More convenient than GP (11.5%) • GP not available (10.5%) • No GP or GP > 25 miles away (9%) • Other (2%)
(Porro, Monzani et al. 2013) [82] Italy Cross-sectional survey administered via structured interview *N* = 583 NIH = 4	Age: Not reported; Gender: Not reported Triage: Not specified (patients categorized by “appropriateness:” 1) appropriate (i.e., sudden health problem, 2) inappropriate (i.e., long-standing problem), 3) hybrid (i.e., long-standing problem that suddenly re-emerged/worsened))	• Possibility to obtain all necessary examination at the same time (*N* = 232) • Fastest solution for complaint (*N* = 187) • Closest solution (*N* = 169) • Suggested by a pharmacist (*N* = 99) • Could not wait for family doctor visiting hours (*N* = 97) • Suggested by relatives/friends (*N* = 60) • Cheapest solution (*N* = 12)
(Rajpar, Smith et al. 2000) [44] UK Semi-structured questionnaire completed via interviews *N* = 102 (*N* = 54 ED only) NIH = 4	ED Patients: Mean age: 27.9 yr; Gender: males = 26, females = 28 Triage: Not specified (patients with primary care problems were defined as “those with non-emergency problems that could be managed in an average local GP surgery and triaged not to require treatment within two hours”)	• Stated “GP was closed” (50.0%) • Perceived severity of problem (22.2%) • Did not want to disturb their GP (11.1%) • Wanted second opinion (7.4%) • Perceived wait time in ED shorter than at GP (5.6%) • Perceived that facility and investigations better at ED (3.7%)
(Rieffe, Oosterveld et al. 1999) [84] Netherlands Cross-sectional questionnaire *N* = 430 NIH = 4	Mean age: 31.0 ± 15.1 yr; Gender: males = 280, females = 150 Triage: Not specified (no-urgent patients determined by whether their condition lasted > 24 h, and according to a classification scheme created by ED experts and applied by a medical student)	• 21 Motive Scales evaluating 63 different reasons for ED attendance (proportion of patients responding not reported, only mean scores); overall, motives primarily related to financial means and/or the preference of the expertise and facilities of ED
(Thomson, Kohli et al. 1995) [60] UK Cross-Sectional Survey *N* = 245 NIH = 4	Mean age: 28.5 yr; Gender: males = 162, females = 83 Triage: Not specified (non-urgent patients determined to “not require immediate attention by a physician and could wait as necessary” and who had attended the ED without previously contacted their GP)	• Easier geographical access (15%) • Convenience-related to timing (24%) • GPs perceived inability to treat disorder (59%) • Other (3%)
(Galanis, Siskou et al. 2019) [62] Greece Cross-sectional survey *N* = 307 NIH = 2	Mean age: 50.4 yr ± 19.8 yr; Gender: not reported Triage: Hospital Urgencies Appropriateness Protocol (HUAP)	• Patients had more confidence in hospital rather than primary care services/patients expected better care in EDs (46.6%) • Patients’ residence was closer to the hospital (44.6%) • Patients needed diagnostic tests (X-rays, laboratory tests, etc.) (31.6%) • Patients were not aware whether an out-of-hospital emergency health service was at their disposal or its contact details (telephone number or address) (27%) • Long waiting lists for hospital outpatient consultation (20.8%) • Long waiting lists for appointments with non-hospital specialists (19.2%) • Long waiting lists for primary care consultation (with contracted physicians or in health centers) (16.9%) • Patients’ family prompted them to the EDs (16.9%) • No primary care physician had been assigned to the patient (e.g., family doctor) (16.3%) • Lack of a (primary care) physician in the public health system (14.3%) • Inability to contact primary care services (13%) • Patient did not trust their primary care physician (10.1%)

*AED* Accident and Emergency Room, *ED* Emergency Department, *ER* Emergency Room, *GP* General Practitioner

In examining five-year intervals, 11 studies were published in 1995–1999, 8 studies in 2000–2004, 18 studies in 2005–2009, 23 studies in 2010–2014, 24 studies in 2015–2019 and 9 in 2020–2021. Studies were published across 16 different countries, the majority of which originated from just five countries (64.5%): United States (*N* = 20), United Kingdom (*N* = 18), Canada (*N* = 8), Australia/New Zealand (*N* = 7), and the Netherlands (*N* = 6). Among the remaining 33 studies, 18 originated from Europe, 3 from South America, 5 from Asia, 6 from the Middle East, and 2 from Africa. Some studies evaluated reasons for using the ED among patients with all types of medical severity. However, for this review, only reasons for attending were collected on non-urgent patients. The total number of patients included in the studies used for the review (excluding review papers) was 49,238. Approximately one quarter of studies had samples with either less than 100 patients (28.8%), or more than 500 patients (26.6%); the bulk of studies (44.6%) recruited between 100 and 500 patients. Fifteen studies (16.7%) did not provide information on the sex of patients (*N* = 7730 total patients). Among the 75 studies that reported sex ratios, there were 21,044 males (50.2%) and 20,864 females (49.8%) in total. A wide variety of formal triage classification systems were used in 39 studies to assign a severity and urgency of patients’ presenting complaints (Tables 2 and 3). A total of 44 studies did not specify which triage system was used and instead reported that “non-urgent” patients were recruited for participation. In seven studies, a 3-, 4-, or 5-level triage system was described but it was not formally named. The most commonly used triage system was the Canadian Triage and Acuity Scale (CTAS; *N* = 12) and this was used within and outside of Canada.

### Themes

After comparing and contrasting major reasons for non-urgent ED use among studies, a total of seven major themes were identified:

1. Need to be Risk Averse with Respect to the Health Issue
2. Knowledge and Awareness of Alternative Sources of Care
3. Dissatisfaction with Primary Care Provider (PCP) (Subthemes: availability, competence, preference);
4. Satisfaction with ED (Subthemes: quality care, access to ED-specific services);
5. ED Accessibility and Convenience Resulting in Low Access Burden
6. Referred to the ED by Others (Subthemes: health care professionals, non-health care professionals); and
7. Relationships between Patients and Health Care Providers

Each theme and sub-theme will be described. Some patients reported that they had no specific reason for attending the ED [32, 35, 36, 63, 79, 81]. Several studies stated that there were “other, unspecified reasons” reported by patients; however, there were no further details provided [19, 28, 32–36, 38, 42, 47, 51, 60, 61, 64, 65, 69, 76, 77, 79, 81, 91, 103].

### Theme 1: Need to be risk averse with respect to the health issue

One of the primary reasons reported in the literature for presentation at an ED was the tendency of patients to be risk averse in terms of their health issue. There was a self-perceived sense of severity or urgency to their medical matters, despite that their presenting complaints were deemed non-urgent [16–54, 85–90]. Many patients described having feelings of anxiety, uncertainty or significant concern about their health problem [22, 45, 65, 70, 88, 92–95]. Often patients had experienced pain or other discomforts which impacted their function and they desired immediate relief [22, 42, 45, 70–73, 88, 93, 94, 96, 97]. In some cases, they had attempted self-treatment at home, without good effect [73, 86, 96, 98], or had sought out primary care without resolve [21, 42, 53, 66, 74, 75, 88, 92]. Even when patients knew their condition was non-urgent, they still wanted reassurance, advice, or a second opinion [26, 33, 37, 43, 44, 48, 53, 55, 56, 64, 69, 72, 88, 94, 97]. One study found that patients had a self-perceived inability to cope [88]. The ability to leave the ED with a confirmed diagnosis or answer to their health problem (attestation) was particularly helpful in mitigating their fears of a real emergency [54, 55, 100, 102, 103].

### Theme 2: Knowledge and awareness of alternative sources of care

Studies reported that some ED patients had limited knowledge and awareness of alternative sources of medical care. They were unaware or unsure of the differences between services [16, 27, 33, 35, 43, 51, 52, 55, 62, 66, 76, 78, 87, 89, 95] or simply had not considered going to their PCP [64, 90]. Patients reported that they believed the ED was the only and most appropriate option [22, 24, 31, 34, 40, 45, 54, 61, 63–69, 86, 90, 91]. Some people did not want to bother their PCP and did not feel it was necessary to seek primary care first [35, 43, 44, 53, 55, 77, 79].

### Theme 3: Dissatisfaction with primary care provider

One of the most prevalent themes was related to patients’ dissatisfaction with primary care services. Within this theme there were three sub-themes: availability, competency, and preference.

#### Sub-theme 3a: Availability

For a variety of reasons, patients reported extreme difficulty in finding an available PCP [18, 32–35, 38, 41, 42, 45, 46, 52–55, 61, 62, 72, 89, 92, 94]. They could not obtain a PCP appointment at all [17, 20, 21, 27, 28, 33, 35, 36, 66, 69, 79, 80, 92, 93, 98, 99], or they could not obtain an appointment that did not interfere with work/school [46, 54, 66, 73, 93, 96, 101], or childcare [96]. There were significant issues obtaining care after hours or as a result of limited hours provided by the PCP [16, 19, 22, 28, 33, 35–37, 41, 44, 47–50, 54, 56, 57, 66, 72, 74, 85, 87, 92, 94, 97–99, 101–103]. Some patients were not registered with a PCP [18, 25, 27, 29, 30, 35, 36, 43, 49, 54, 55, 58, 61, 62, 64, 66, 77] or there were no primary care options at all [26, 28, 45, 46, 54, 62, 66, 70, 90, 92, 96, 97, 102]. A large majority of patients felt that it took too long to wait for an appointment with the PCP, even if they were successful in scheduling one [20, 21, 24, 25, 36, 37, 48, 49, 53, 56, 58–60, 62, 74, 81, 82, 88, 91, 92, 99–101]. Finally, some studies reported general PCP inconvenience as a reason for non-urgent ED use, although it was not further described [53, 54, 69].

#### Sub-theme 3b: Competency

A large number of patients reported dissatisfaction with their PCP’s ability to handle their ED concern, which was related to their perceived inadequacy and incompetency. Patients reported feeling dissatisfied with their PCP/staff and even discussed mistrusting them [16, 19, 25, 27, 32, 43, 45, 47, 52, 53, 57, 66, 69, 76, 77, 87, 89, 90, 104]. Some patients thought that their PCP was not capable, could not help them or did not have the necessary resources required to handle their presenting complaint [17, 25, 37, 48, 53, 55–62].

#### Sub-theme 3c: Preference

In certain health systems, PCP’s operate within a ‘cooperative’ whereby a team of physicians care for a roster of patients. Some patients indicated that, as a result of this model, they had an inconsistent PCP each time they made an appointment and this was less desirable to them [43, 56]. Other barriers to primary care included to varying language, culture and communication practices [45, 48, 56, 90].

### Theme 4: Satisfaction with ED

Satisfaction with the ED was a highly cited reason for attending non-urgently. This theme included two sub-themes related to benefits of the ED, namely quality of care and access to ED-specific services.

#### Sub-theme 4a: Quality care

A large number patients reported that the ED afforded them superior care, beyond what could be obtained in primary care. Patients believed ED care was of higher quality and as such, they had greater trust and confidence in the ED [18, 19, 21, 22, 27, 30–33, 37, 39, 41–48, 54, 56, 58, 61–65, 74–79, 83, 84, 87, 94, 97, 98, 103]. Investigations were perceived to be more thorough [21, 35, 77, 78, 92, 103], with all resources available in one location [18, 22, 26, 28, 33, 37, 40, 45, 48, 56, 66, 82, 86, 93–95, 97, 98, 100]. Many patients reported that this was their preferred medical setting, that they were familiar with it, and had previous positive experiences in the ED [16, 22, 26, 30, 36, 42, 43, 45, 53, 56, 63, 68, 86–88, 90, 92, 94, 97].

#### Sub-theme 4b: Access to ED-specific services

The ED is unique in that it provides patients access to a wide variety of resources necessary for assessing, monitoring, managing and treating conditions for most medical problems. Patients reported attending the ED non-urgently to gain access to these ED-specific services they could not otherwise access through a PCP either in a timely fashion, or all in one visit [29, 43, 47, 49, 54, 55, 66, 70–73, 84, 95]. These included access to diagnostic investigations (e.g., imaging, bloodwork) [18, 25, 34, 42, 51, 56, 59, 62, 71–74, 79, 80, 93–95, 99, 101, 103], access to medication [22, 52, 73, 81, 93, 103], access to specialists [55, 62, 66, 73, 76, 100, 102, 103], or a pathway to hospital admission which they perceived was necessary [25, 40, 55, 59, 65, 66, 72, 103].

### Theme 5: ED accessibility and convenience resulting in low access burden

Compared to other primary care options, the ease, accessibility and convenience offered in the ED provided patients with a low burden of access to medical care [17, 18, 21, 22, 26, 30, 31, 34–38, 43, 47, 51, 59, 61, 63, 65, 68, 70, 72, 76, 79, 85, 87, 90, 92, 105]. Patients reported that it saved them time and overall the wait was short in order to receive help [18, 19, 26, 27, 30–33, 39, 42–44, 55, 60, 61, 64, 66, 81, 82, 94, 95, 99, 100, 103, 105]. Some patients faced transportation barriers getting to their PCP so it was easier to access the ED [54, 55, 89, 96]. Similarly, a great number of patients reported geographical proximity to the ED as a motivating factor for attending non-urgently [16, 19, 22, 27, 30, 32, 35, 36, 41–43, 45–48, 53–57, 60–62, 64, 66, 72, 74, 75, 77, 81–83, 92, 94, 95]. Other convenience factors, such as not requiring an appointment [18, 22, 33, 37, 43, 45, 48, 53, 56, 58, 76, 95] and unrestricted availability (open day and night) [25, 40, 72] were cited as important indicators for ED use. For patients seeking care where medical insurance coverage may be problematic, EDs were often sought out for relief of any financial burden [19, 20, 22, 24, 30, 32, 34, 35, 39, 41, 45, 46, 48, 51, 53, 54, 57, 65, 68, 72, 75, 78, 82, 84, 87, 89]. In a small number of studies, the reason was circumstantial. For example, patients reported being on vacation or were from out of town [56, 58] whereas others just happened to be at the hospital for an unrelated reason [53, 81].

### Theme 6: Referred to the ED by others

Patients were often referred by others to attend the ED for their problem; there were two types of referrals discussed, those made by health care professionals and those made by non-health care professionals.

#### Sub-theme 6a: Health care professionals

Patients reported being told, although this was not verified by most studies, to go to the ED by their PCP [17, 19, 20, 28, 31–36, 41, 42, 50–52, 54, 56, 58, 61, 63, 67, 69, 71, 72, 74, 78, 80, 89, 91, 98, 100, 104], or by non-PCP clinic staff (e.g., medical secretaries) [17, 19, 33, 80, 90, 99, 104]. In some cases, patients reported attending because they believed their PCP would send them anyway, even if they had not contacted them at all [34, 38, 43, 56, 91]. Patients stated they had attended on the suggestion of non-physician health care providers [17, 19, 31–33, 51, 69, 72, 78, 96, 104], a health line [51] or a pharmacist [82].

#### Sub-theme 6b: Non-health care professionals

Patients stated that non-health care professionals referred them to the ED [26, 27, 30, 43, 59, 61, 63, 73, 91]. For example, family, friends, and others in patients’ social network were influential in telling them they should go to the ED [22, 33, 35, 38, 41, 42, 45, 62, 64, 70, 72, 82, 83, 86, 88, 90, 96, 105]. In two studies, patients stated their reason for attending was based on influences by the media (i.e., advertisements) [53, 97]. For others, patients were specifically directed to the ED by their employer [72, 105] or by the police [71].

### Theme 7: Relationships between patients and health care providers

There are often dynamic interactions or relationships between patients and health care providers. In certain groups and geographical regions, use of the ED was an automatic, habitual behavioural or cultural practice shared by many patients [26, 33, 42, 45, 47, 48, 51, 52, 56, 66, 76, 83, 87]. For hospital staff or members of their family, the ED was a logical place to attend given their proximity to place of employment; the relationships these patients had with the ED (and the health care system at large) facilitated its use [19, 32, 42, 46, 47]. Attending the ED, even non-urgently, also made ‘sense’ for those who were currently (or previously) receiving treatment from that hospital already [19, 22, 27, 32, 34, 36, 45, 51, 65, 72, 78, 79, 95]. Conversely, for others, the ED acted as a place of anonymity because no relationship existed. The possibility of obtaining medical care from a doctor they did not know [37, 48, 56] or from someone of the same or opposite sex [48] was appealing.

## Discussion

### Summary of results

The aim of this study was to conduct an integrative review of the scientific literature to explore patient-reported reasons for using the ED non-urgently. The studies included for review reported that attending the ED was an intentional decision based on several influential factors. Seven main themes were identified: 1) Need to be risk averse with respect to the health issue; 2) Knowledge and awareness of alternative sources of care; 3) Dissatisfaction with PCP (Subthemes: availability, competence, preference); 4) Satisfaction with ED (Subthemes: quality care, access to ED-specific services); 5) ED accessibility and convenience resulting in low access burden; 6) Referred to the ED by others (Subthemes: health care professionals, non-health care professionals); and 7) Relationships between patients and health care providers. For many patients, there was a very clear problem which needed to be addressed, whether it was physical, psychological, or social. After weighing several options, from their perspective their need was real and the ED as an option for care was rational and justified, not just their last resort.

### Context of other research

The results reported here are well-aligned with other reviews [5–7], but also extend the current knowledge of the subject by providing a comprehensive synthesis of all extant literature of reasons for non-urgent ED use. Recently, O’Cathain et al. [5] examined non-urgent ED use using a ‘realist review.’ Building on earlier reviews [6, 7, 106], they performed an updated literature search to the end of January 2017. They compiled and compared results from 29 quantitative studies, existing health behaviour theories, and 32 qualitative studies. Our integrative review was able to validate and supplement the ten program theories and six mechanisms of decision-making as described by O’Cathain et al. [5] with a larger compilation of studies. With respect to program theories, we did not uncover the theme of ‘fear of consequences when responsible for others’ found by these authors. This theme potentially relates to individuals’ responsibility to care for children, and we did not include studies on the pediatric population. Five mechanisms of decision-making were described by O’Cathain et al. [5] and shared with our integrative review (i.e., the need to be risk averse with respect to a health issue, ED accessibility and convenience resulting in low access burden, satisfaction with ED, dissatisfaction with PCP, and referral to ED by others). However, they reported that there was either limited or no support at all from the quantitative literature with respect to experiences of past traumatic events, anxiety, stress, coping, and need for immediate pain relief. In contrast, we found significant support for these reasons within the quantitative literature included here for review. Further, two themes not emphasized by O’Cathain et al. [5] were found to be highly influential in this review (i.e., Knowledge and awareness of alternative sources of care and Relationships between patients and health care providers). The additional studies incorporated in this integrative review (*N* = 60), not previously captured in other reviews, serve to both validate and enhance our previous understanding of the context surrounding decision-making for non-urgent ED.

### Clinical implications

While there is the wealth of knowledge on this topic, the majority of studies were published from highly resourced nations (i.e., USA, UK, Canada, Australia); as such, the results should be considered in light of this context. For example, Canada has a publicly funded healthcare system which contrasts with the private health care model utilized in the United States, and various two-tiered systems adopted in Europe and Australia. American studies have reported financial barriers to primary care as a common reason for attending the ED [107]. Financial barriers are not particularly relevant to individuals from nations with public or semi-public health systems since they, in part, have a reduced (direct) financial responsibility for medical care. Our understanding of reasons for non-urgent ED use in less resourced nations is currently limited.

The results from this review suggest that ED patients are heterogenous and that many factors influence their decision-making. Considering the complexity of patients that EDs care for, treating them as a single entity may be problematic. Thus, a multi-pronged approach may be required to limit excessive non-urgent visits. For example, simply redirecting non-urgent patients to other settings has been shown not to be wholly effective [108]. Instead, ensuring health care providers (at both PCP clinics and ED) understand how and why patients make decisions may help to provide insight and direct patient education. Health education should be explicitly and intentionally embedded in all ED health care provider roles [109]. This involves communicating, managing knowledge, mitigating errors, and supporting decision-making [109]. Research suggests that basic educational expertise, fundamental knowledge and reasoning, as well as emotional self-regulation are all critical components of health [110]. Thus, education is a social determinant of health which can potentially impede or enhance patients’ health [110]. Routinely educating patients on the role of the ED, as well as alternatives in the community, is a critical aspect of improving the public’s health.

This review found that many patients were anxious, uncertain, or fearful of their health problem. They had decreased ability to manage their discomforts and some reported the inability to cope. Guidance and support should be provided to patients with respect to managing recurring symptoms which may be directly or indirectly (e.g., anxiety, stress) related the presenting condition. Discharge teaching could include problem solving techniques for decision-making (e.g., accessing information) as well as self-management strategies (e.g., pain relief). While these “common sense” strategies may be commonplace among health care providers, it should not be assumed they are shared with lay persons. A recent systematic review highlighted and confirmed the disparity in patients’ and clinicians’ mutual understanding [111]. The authors examined the effectiveness of different methods of providing discharge instructions in the ED and found that communicating discharge instructions verbally may be insufficient; greater success could be achieved with the addition of video or written information [111], or via social media.

Finally, this integrative review demonstrated that there are notable deficiencies in various design and functioning of health care systems, where the literature was drawn. Many patients reported significant issues with accessing primary care, and were dissatisfied as a result. Simultaneously, patients were satisfied with the ED due to increased accessibility and quality of care, thus driving their attendance. This result has been supported by Van den Borg et al. [112] who examined the relationship between attending the ED and accessibility and continuity of primary care among 34 countries (60,991 patients). They found that ED visits had a significant and negative relationship with better primary care accessibility [112]. Systematically improving deficiencies in primary care may reduce non-urgent ED visits. Policy makers and practitioners should reflect and consider the complexities of their given health care environments to adequately design systems which are responsive to patients needs.

### Research implications

There has been a significant amount of inquiry generated on patient-reported reasons for non-urgent ED use. Regardless, there are a few areas that should be targeted for deeper inspection which would assist in filling gaps in the knowledge and addressing certain methodological considerations. Future research should aim to explore, in greater depth, specific themes identified in this review. For example, the role of health knowledge, emotions, beliefs, attitudes, and behaviour response patterns have been indicated as influencing the decision-making process, specifically with respect to perceived severity and urgency of presenting condition. New studies should explicitly evaluate ED users’ health literacy, health-related personal beliefs, stress and coping ability using validated outcome measures. This approach has received little to no attention in the literature. Psychosocial factors (e.g., stress, coping) have been explored in only a dearth of studies, largely as an afterthought to the primary objective [2]. Linking this subjective data to large, objective administrative health data could provide greater context than simple patient-reported reasons. Researchers should endeavour to use standardized criteria to evaluate triage acuity, when possible, and to fully describe their patient population and geographic region for accurate interpretation of results and comparisons with others.

### Limitations

This integrative review is not without its own limitations. The strict inclusion and exclusion criteria may have limited some articles from being included (e.g., all non-English studies). Further, specific populations (e.g., ambulance riders, pediatrics, specific presenting complaints, frequent ED users) have been cited as using the ED non-urgently but studies focused specifically on such subgroups were excluded from this review. This was intentional in an attempt to create a more homogenous sample for review. These ED subgroups may contribute unique results which could be informative to this topic. Nevertheless, results were drawn from a very large pool of general ED population studies. Finally, integrative reviews have the potential to suffer from lack of rigor given the process of combining diverse, complex methodologies [9]. The methods described herein were conducted using an iterative coding process by two individuals following well-cited, formulated guidance [9, 13].

## Conclusion

This integrative review summarized over 30 years of research evidence on patient-reported reasons for non-urgent ED use. It was conducted using a rigorous systematic methodology and data analysis in accordance with widely accepted reporting criteria. The inclusion of both qualitative and quantitative studies led to a comprehensive understanding of seven major themes associated with decision-making, namely: Need to be risk averse with respect to the health issue; Knowledge and awareness of alternative sources of care; Dissatisfaction with PCP; Satisfaction with ED; ED accessibility and convenience resulting in low access burden; Referred to the ED by others; and Relationships between patients and health care providers. Future studies should use validated outcome measures to specifically explore the role of complex psychosocial factors driving decision-making including health literacy, health-related personal beliefs, stress and coping ability.

## Supplementary Information

**Additional file 1.**

## Data Availability

The datasets used and/or analysed during the current study are available from the corresponding author on reasonable request.
