# Supplementary material for: An integrative review of adult patient-reported reasons for non-urgent use of the emergency department
Source: BMC Nurs. 2023 Mar 30;22:85. doi: 10.1186/s12912-023-01251-7 (PMC10061911; doi:10.1186/s12912-023-01251-7)
Supplement: Supplementary file 1 — Additional file 1. [file 12912_2023_1251_MOESM1_ESM.docx]

**Excluded References**

Adigun, A. C., Maguire, K., Jiang, Y., Qu, H., & Austin, S. (2019). Urgent Care Center and Emergency Department Utilization for Non-Emergent Health Conditions: Analysis of Managed Care Beneficiaries. Population Health Management, 22(5), 433-439. https://doi.org/10.1089/pop.2018.0138

Backman, A., Blomqvist, P., Lagerlund, M., Carlsson-Holm, E., & Adami, J. (2008). Characteristics of non-urgent patients: cross-sectional study of emergency department and primary care patients. Scandinavian Journal of Primary Health Care, 26(3), 181-187. https://doi.org/10.1080/02813430802095838

Backman, A. S., Blomqvist, P., Lagerlund, M., & Adami, J. (2010, May). Physician assessment of appropriate healthcare level among nonurgent patients. Am J Manag Care, 16(5), 361-368.

Benahmed, N., Laokri, S., Zhang, W. H., Verhaeghe, N., Trybou, J., Cohen, L., De Wever, A., & Alexander, S. (2012, Dec). Determinants of nonurgent use of the emergency department for pediatric patients in 12 hospitals in Belgium. Eur J Pediatr, 171(12), 1829-1837. https://doi.org/10.1007/s00431-012-1853-y

Berry, A., Brousseau, D., Brotanek, J. M., Tomany-Korman, S., & Flores, G. (2008, Nov-Dec). Why do parents bring children to the emergency department for nonurgent conditions? A qualitative study. Ambul Pediatr, 8(6), 360-367. https://doi.org/10.1016/j.ambp.2008.07.001

Booker, M. J., Shaw, A. R., & Purdy, S. (2015, May 19). Why do patients with 'primary care sensitive' problems access ambulance services? A systematic mapping review of the literature. BMJ Open, 5(5), e007726. https://doi.org/10.1136/bmjopen-2015-007726

Booker, M. J., Simmonds, R. L., & Purdy, S. (2014, Jun). Patients who call emergency ambulances for primary care problems: a qualitative study of the decision-making process. Emerg Med J, 31(6), 448-452. https://doi.org/10.1136/emermed-2012-202124

Brousseau, D. C., Nimmer, M. R., Yunk, N. L., Nattinger, A. B., & Greer, A. (2011, Feb). Nonurgent emergency-department care: analysis of parent and primary physician perspectives. Pediatrics, 127(2), e375-381. https://doi.org/10.1542/peds.2010-1723

Capp, R., Kelley, L., Ellis, P., Carmona, J., Lofton, A., Cobbs-Lomax, D., & D'Onofrio, G. (2016, Apr). Reasons for Frequent Emergency Department Use by Medicaid Enrollees: A Qualitative Study. Acad Emerg Med, 23(4), 476-481. https://doi.org/10.1111/acem.12952

Chang, C. F., & Troyer, J. L. (2011). Trends in potentially avoidable hospitalizations among adults in Tennessee, 1998-2006 [Article]. Tennessee medicine : journal of the Tennessee Medical Association, 104(10), 35-38, 45. https://www.scopus.com/inward/record.uri?eid=2-s2.0-84855363776&partnerID=40&md5=9ecf20a9b0718cdb643262abd2e91567

Chen, B. K., Hibbert, J., Xi, C., & Bennett, K. (2015). Travel distance and sociodemographic correlates of potentially avoidable emergency department visits in California, 2006-2010: an observational study. International Journal for Equity in Health, 14(1), 1-8. https://doi.org/10.1186/s12939-015-0158-y

Chin, N. P., Goepp, J. G., Malia, T., Harris, L., & Poordabbagh, A. (2006, Jan). Nonurgent use of a pediatric emergency department: a preliminary qualitative study. Pediatr Emerg Care, 22(1), 22-27. https://doi.org/10.1097/01.pec.0000195756.74328.21

Chmiel, C., Huber, C. A., Rosemann, T., Zoller, M., Eichler, K., Sidler, P., & Senn, O. (2011, May 9). Walk-ins seeking treatment at an emergency department or general practitioner out-of-hours service: a cross-sectional comparison. BMC Health Serv Res, 11, 94. https://doi.org/10.1186/1472-6963-11-94

Colineaux, H., Pelissier, F., Pourcel, L., Lang, T., Kelly-Irving, M., Azema, O., Charpentier, S., & Lamy, S. (2019). Why are people increasingly attending the emergency department? A study of the French healthcare system. Emergency Medicine Journal, 36(9), 548-553. https://doi.org/10.1136/emermed-2018-208333

Corwin, G. S., Parker, D. M., & Brown, J. R. (2016, Sep). Site of Treatment for Non-Urgent Conditions by Medicare Beneficiaries: Is There a Role for Urgent Care Centers? Am J Med, 129(9), 966-973. https://doi.org/10.1016/j.amjmed.2016.03.013

Davis, J. W., Fujimoto, R. Y., Chan, H., & Juarez, D. T. (2010, Oct). Identifying characteristics of patients with low urgency emergency department visits in a managed care setting. Manag Care, 19(10), 38-44.

de Bont, E. G., Loonen, N., Hendrix, D. A., Lepot, J. M., Dinant, G. J., & Cals, J. W. (2015, Oct 7). Childhood fever: a qualitative study on parents' expectations and experiences during general practice out-of-hours care consultations. BMC Fam Pract, 16, 131. https://doi.org/10.1186/s12875-015-0348-0

Deavenport-Saman, A., Lu, Y., Smith, K., & Yin, L. (2016, Feb). Do Children with Autism Overutilize the Emergency Department? Examining Visit Urgency and Subsequent Hospital Admissions. Matern Child Health J, 20(2), 306-314. https://doi.org/10.1007/s10995-015-1830-y

Dinh, M. M., Berendsen Russell, S., Bein, K. J., Chalkley, D. R., Muscatello, D., Paoloni, R., & Ivers, R. (2016, May 10). Statewide retrospective study of low acuity emergency presentations in New South Wales, Australia: who, what, where and why? BMJ Open, 6(5), e010964. https://doi.org/10.1136/bmjopen-2015-010964

Durant, E., & Fahimi, J. (2012, Jul-Sep). Factors associated with ambulance use among patients with low-acuity conditions. Prehosp Emerg Care, 16(3), 329-337. https://doi.org/10.3109/10903127.2012.670688

Farion, K. J., Wright, M., Zemek, R., Neto, G., Karwowska, A., Tse, S., Reid, S., Jabbour, M., Poirier, S., Moreau, K. A., & Barrowman, N. (2015). Understanding Low-Acuity Visits to the Pediatric Emergency Department. PLoS One, 10(6), e0128927. https://doi.org/10.1371/journal.pone.0128927

Fieldston, E. S., Alpern, E. R., Nadel, F. M., Shea, J. A., & Alessandrini, E. A. (2012, Mar). A qualitative assessment of reasons for nonurgent visits to the emergency department: parent and health professional opinions. Pediatr Emerg Care, 28(3), 220-225. https://doi.org/10.1097/PEC.0b013e318248b431

Freed, G., Gafforini, S., & Carson, N. (2015, Aug). Age-related variation in primary care-type presentations to emergency departments. Aust Fam Physician, 44(8), 584-588.

Freed, G. L., Allen, A. R., Turbitt, E., Nicolas, C., & Oakley, E. (2016, Apr). Parent perspectives and reasons for lower urgency paediatric presentations to emergency departments. Emerg Med Australas, 28(2), 211-215. https://doi.org/10.1111/1742-6723.12544

Fusco, M., Buja, A., Furlan, P., Casale, P., Marcolongo, A., Baldovin, T., Bertoncello, C., & Baldo, V. (2014, Sep-Oct). Older adults in Emergency Department: management by clinical severity at triage. Ann Ig, 26(5), 409-417. https://doi.org/10.7416/ai.2014.2000

Gandhi, S. O., Grant, L. P., & Sabik, L. M. (2014, Oct). Trends in nonemergent use of emergency departments by health insurance status. Med Care Res Rev, 71(5), 496-521. https://doi.org/10.1177/1077558714541481

Garcia, T. C., Bernstein, A. B., & Bush, M. A. (2010, May). Emergency department visitors and visits: who used the emergency room in 2007? NCHS Data Brief(38), 1-8.

Goepp, J. G., Chin, N. P., Massad, J., & Edwards, L. A. (2004, Nov). Pediatric emergency department outreach: solving medical problems or revealing community solutions? J Health Care Poor Underserved, 15(4), 522-529. https://doi.org/10.1353/hpu.2004.0060

Grant, R., Ramgoolam, A., Betz, R., Ruttner, L., & Green, J. J. (2010, Oct 1). Challenges to accessing pediatric health care in the Mississippi delta: a survey of emergency department patients seeking nonemergency care. J Prim Care Community Health, 1(3), 152-157. https://doi.org/10.1177/2150131910380727

Guimaraes, D. S., Jr., Soares, E. J., Junior, G. F., & Medeiros, D. D. (2015, Feb 18). Attributes and circumstances that induce inappropriate health services demand: a study of the health sector in Brazil. BMC Health Serv Res, 15, 65. https://doi.org/10.1186/s12913-015-0728-0

Gundlapalli, A. V., Jones, A. L., Redd, A., Ying, S. U. O., Pettey, W. B. P., Mohanty, A., Brignone, E., Gawron, L., Vanneman, M., Samore, M. H., & Fargo, J. D. (2017). Characteristics of the Highest Users of Emergency Services in Veterans Affairs Hospitals: Homeless and Non-Homeless..."International Conference on Informatics, Management, and Technology in Healthcare," Athens, Greece, 2017. Studies in Health Technology & Informatics, 238, 24-27. https://doi.org/10.3233/978-1-61499-781-8-24

Hassankhani, H., Soheili, A., Vahdati, S. S., Amin Mozaffari, F., Wolf, L. A., & Wiseman, T. (2019). "Me First, Others Later" A focused ethnography of ongoing cultural features of waiting in an Iranian emergency department. International Emergency Nursing, 47, N.PAG-N.PAG. https://doi.org/10.1016/j.ienj.2019.100804

Hjälte, L., Suserud, B. O., Herlitz, J., & Karlberg, I. (2007, Jun). Why are people without medical needs transported by ambulance? A study of indications for pre-hospital care. Eur J Emerg Med, 14(3), 151-156. https://doi.org/10.1097/MEJ.0b013e3280146508

Hong Choon, O., Wai Leng, C., Yan, G., Ling, T., Siang Hiong, G., Mohan, T., Oh, H. C., Chow, W. L., Gao, Y., Tiah, L., & Goh, S. H. (2020). Factors associated with inappropriate attendances at the emergency department of a tertiary hospital in Singapore. Singapore Medical Journal, 61(2), 75-80. https://doi.org/10.11622/smedj.2019041

Houston, A. M., & Pickering, A. J. (2000, Dec). 'Do I don't I call the doctor': a qualitative study of parental perceptions of calling the GP out-of-hours. Health Expect, 3(4), 234-242. https://doi.org/10.1046/j.1369-6513.2000.00109.x

Hugenholtz, M., Bröer, C., & van Daalen, R. (2009, Mar). Apprehensive parents: a qualitative study of parents seeking immediate primary care for their children. Br J Gen Pract, 59(560), 173-179. https://doi.org/10.3399/bjgp09X394996

Jacob, S. L., Jacoby, J., Heller, M., & Stoltzfus, J. (2008, Apr-Jun). Patient and physician perspectives on ambulance utilization. Prehosp Emerg Care, 12(2), 176-181. https://doi.org/10.1080/10903120701710058

Johnson, P. J., Ghildayal, N., Ward, A. C., Westgard, B. C., Boland, L. L., & Hokanson, J. S. (2012). Disparities in Potentially Avoidable Emergency Department (ED) Care: ED Visits for Ambulatory Care Sensitive Conditions. Medical Care, 50(12), 1020-1028. https://doi.org/10.1097/MLR.0b013e318270bad4

Kallestrup, P., & Bro, F. (2003, Jan). Parents' beliefs and expectations when presenting with a febrile child at an out-of-hours general practice clinic. Br J Gen Pract, 53(486), 43-44.

Kawakami, C., Ohshige, K., Kubota, K., & Tochikubo, O. (2007, Jul 27). Influence of socioeconomic factors on medically unnecessary ambulance calls. BMC Health Serv Res, 7, 120. https://doi.org/10.1186/1472-6963-7-120

Kirkby H, R. L. (2012). Inappropriate 999 calls: an online pilot survey. Emerg Med J, 29.

Lehm, K. K., Andersen, M. S., & Riddervold, I. S. (2017). Non-urgent Emergency Callers: Characteristics and Prognosis. Prehospital Emergency Care, 21(2), 166-173. https://doi.org/10.1080/10903127.2016.1218981

Leporatti, L., Ameri, M., Trinchero, C., Orcamo, P., & Montefiori, M. (2016, May). Targeting frequent users of emergency departments: Prominent risk factors and policy implications. Health Policy, 120(5), 462-470. https://doi.org/10.1016/j.healthpol.2016.03.005

Lynch, B., Fitzgerald, A. P., Corcoran, P., Buckley, C., Healy, O., & Browne, J. (2019). Drivers of potentially avoidable emergency admissions in Ireland: an ecological analysis. BMJ Quality & Safety, 28(6), 438-448. https://doi.org/10.1136/bmjqs-2018-008002

Malone, R. E. (1998, Oct). Whither the almshouse? Overutilization and the role of the emergency department. J Health Polit Policy Law, 23(5), 795-832. https://doi.org/10.1215/03616878-23-5-795

McHale, P., Wood, S., Hughes, K., Bellis, M. A., Demnitz, U., & Wyke, S. (2013, Dec 13). Who uses emergency departments inappropriately and when - a national cross-sectional study using a monitoring data system. BMC Med, 11, 258. https://doi.org/10.1186/1741-7015-11-258

Mostajer Haqiqi, A., Bedos, C., & Macdonald, M. E. (2016, Oct). The emergency department as a 'last resort': why parents seek care for their child's nontraumatic dental problems in the emergency room. Community Dent Oral Epidemiol, 44(5), 493-503. https://doi.org/10.1111/cdoe.12239

Mowbray, F. I., Omar, A. E., Pfaff, K., & El-Masri, M. M. (2019). Exploring the factors associated with non-urgent emergency department utilisation for mental health care. Journal of Research in Nursing, 24(8), 663-674. https://doi.org/10.1177/1744987119845020

Naouri, D., Ranchon, G., Vuagnat, A., Schmidt, J., El Khoury, C., & Yordanov, Y. (2020). Factors associated with inappropriate use of emergency departments: findings from a cross-sectional national study in France. BMJ Quality & Safety, 29(6), 449-464. https://doi.org/10.1136/bmjqs-2019-009396

Neill, S. J., Jones, C. H., Lakhanpaul, M., Roland, D. T., & Thompson, M. J. (2016, Mar). Parents' help-seeking behaviours during acute childhood illness at home: A contribution to explanatory theory. J Child Health Care, 20(1), 77-86. https://doi.org/10.1177/1367493514551309

Niska, R., Bhuiya, F., & Xu, J. (2010, Aug 6). National Hospital Ambulatory Medical Care Survey: 2007 emergency department summary. Natl Health Stat Report(26), 1-31.

O'Cathain, A., Knowles, E., Maheswaran, R., Pearson, T., Turner, J., Hirst, E., Goodacre, S., & Nicholl, J. (2014). A system-wide approach to explaining variation in potentially avoidable emergency admissions: national ecological study. BMJ Quality & Safety, 23(1), 47-55. https://doi.org/10.1136/bmjqs-2013-002003

O'Halloran, S. M., & Heaf, D. P. (1989, Aug). Recurrent accident and emergency department attendance for acute asthma in children. Thorax, 44(8), 620-626. https://doi.org/10.1136/thx.44.8.620

O'Keeffe, C., Mason, S., Jacques, R., & Nicholl, J. (2018). Characterising non-urgent users of the emergency department (ED): A retrospective analysis of routine ED data. PLoS One, 13(2), e0192855. https://doi.org/10.1371/journal.pone.0192855

Olsson, M., & Hansagi, H. (2001, Nov). Repeated use of the emergency department: qualitative study of the patient's perspective. Emerg Med J, 18(6), 430-434. https://doi.org/10.1136/emj.18.6.430

Panahpour Eslami, N., Nguyen, J., Navarro, L., Douglas, M., & Bann, M. (2020, Aug 24). Factors associated with low-acuity hospital admissions in a public safety-net setting: a cross-sectional study. BMC Health Serv Res, 20(1), 775. https://doi.org/10.1186/s12913-020-05456-3

Pennycook, A. G., Makower, R. M., & Morrison, W. G. (1991). Use of the emergency ambulance service to an inner city accident and emergency department--a comparison of general practitioner and '999' calls. Journal of the Royal Society of Medicine, 84(12), 726-727. https://search.ebscohost.com/login.aspx?direct=true&db=cin20&AN=104747198&site=ehost-live

Philips, H., Remmen, R., De Paepe, P., Buylaert, W., & Van Royen, P. (2010, Nov 15). Out of hours care: a profile analysis of patients attending the emergency department and the general practitioner on call. BMC Fam Pract, 11, 88. https://doi.org/10.1186/1471-2296-11-88

Pileggi, C., Bianco, A., Di Stasio, S. M., & Angelillo, I. F. (2004, Jun). Inappropriate hospital use by patients needing urgent medical attention in Italy. Public Health, 118(4), 284-291. https://doi.org/10.1016/j.puhe.2003.06.002

Posocco, A., Scapinello, M. P., De Ronch, I., Castrogiovanni, F., Lollo, G., Sergi, G., Tomaselli, I., Tonon, L., Solmi, M., Pescador, D., Battistuz, E., Traversa, S., Zambianco, V., & Veronese, N. (2018). Role of out of hours primary care service in limiting inappropriate access to emergency department. Internal & Emergency Medicine, 13(4), 549-555. https://doi.org/10.1007/s11739-017-1679-8

Rocovich, C., & Patel, T. (2012). Emergency department visits: Why adults choose the emergency room over a primary care physician visit during regular office hours? World J Emerg Med, 3(2), 91-97. https://doi.org/10.5847/wjem.j.issn.1920-8642.2012.02.002

Rutten, M., Vrielink, F., Smits, M., & Giesen, P. (2017, May 12). Patient and care characteristics of self-referrals treated by the general practitioner cooperative at emergency-care-access-points in the Netherlands. BMC Fam Pract, 18(1), 62. https://doi.org/10.1186/s12875-017-0633-1

Sancton, K., Sloss, L., Berkowitz, J., Strydom, N., & McCracken, R. (2018, Aug). Low-acuity presentations to the emergency department: Reasons for and access to other health care providers before presentation. Can Fam Physician, 64(8), e354-e360.

Scapinello, M. P., Posocco, A., De Ronch, I., Castrogiovanni, F., Lollo, G., Sergi, G., Tomaselli, I., Tonon, L., Solmi, M., Traversa, S., Zambianco, V., & Veronese, N. (2016, Sep). Predictors of emergency department referral in patients using out-of-hours primary care services. Health Policy, 120(9), 1001-1007. https://doi.org/10.1016/j.healthpol.2016.07.018

Schappert, S. M. (1995, Oct-Dec). The urgency of visits to hospital emergency departments: data from the National Hospital Ambulatory Medical Care Survey (NHAMCS), 1992. Stat Bull Metrop Insur Co, 76(4), 10-19.

Searing, L. M., & Cantlin, K. A. (2016, Mar-Apr). Nonurgent Emergency Department Visits by Insured and Uninsured Adults. Public Health Nurs, 33(2), 93-98. https://doi.org/10.1111/phn.12238

Seeger, I., Kreienmeyer, L., Hoffmann, F., & Freitag, M. H. (2019). Cross-sectional study in an out-of-hours primary care centre in northwestern Germany - patient characteristics and the urgency of their treatment [Article]. BMC Family Practice, 20(1), Article 41. https://doi.org/10.1186/s12875-019-0929-4

Stewart, M. C., Savage, J. M., Scott, M. J., & McClure, B. G. (1989, Apr). Primary medical care in a paediatric accident and emergency department. Ulster Med J, 58(1), 29-35.

Toloo, G., Bahl, N., Lim, D., FitzGerald, G., Wraith, D., Chu, K., Kinnear, F. B., Aitken, P., & Morel, D. (2020). General practitioner‐type patients in emergency departments in metro North Brisbane, Queensland: A multisite study. Emergency Medicine Australasia, 32(3), 481-488. https://doi.org/10.1111/1742-6723.13447

Toloo, G. S., FitzGerald, G. J., Aitken, P. J., Ting, J. Y., McKenzie, K., Rego, J., & Enraght-Moony, E. (2013, Jun). Ambulance use is associated with higher self-rated illness seriousness: user attitudes and perceptions. Acad Emerg Med, 20(6), 576-583. https://doi.org/10.1111/acem.12149

Unwin, M., Crisp, E., Stankovich, J., McCann, D., & Kinsman, L. (2020). Socioeconomic disadvantage as a driver of non-urgent emergency department presentations: A retrospective data analysis. PLoS One, 15(4), e0231429. https://doi.org/10.1371/journal.pone.0231429

Weiss, A. L., D'Angelo, L. J., & Rucker, A. C. (2014, Apr). Adolescent use of the emergency department instead of the primary care provider: who, why, and how urgent? J Adolesc Health, 54(4), 416-420. https://doi.org/10.1016/j.jadohealth.2013.09.009

Williams, A., O'Rourke, P., & Keogh, S. (2009, Oct). Making choices: why parents present to the emergency department for non-urgent care. Arch Dis Child, 94(10), 817-820. https://doi.org/10.1136/adc.2008.149823

Williams, J. (2013). The prevalence of non-urgent 999 calls: service users' perspectives. Journal of Paramedic Practice, 5(5), 276-277. https://doi.org/10.12968/jpar.2013.5.5.276

Wolinsky, F. D., Liu, L., Miller, T. R., An, H., Geweke, J. F., Kaskie, B., Wright, K. B., Chrischilles, E. A., Pavlik, C. E., Cook, E. A., Ohsfeldt, R. L., Richardson, K. K., Rosenthal, G. E., Wallace, R. B., Wolinsky, F. D., Liu, L., Miller, T. R., An, H., Geweke, J. F., & Kaskie, B. (2008). Emergency department utilization patterns among older adults. Journals of Gerontology Series A: Biological Sciences & Medical Sciences, 63(2), 204-209. https://doi.org/10.1093/gerona/63.2.204

Woolfenden, S., Ritchie, J., Hanson, R., & Nossar, V. (2000, Apr). Parental use of a paediatric emergency department as an ambulatory care service. Aust N Z J Public Health, 24(2), 204-206. https://doi.org/10.1111/j.1467-842x.2000.tb00144.x

Xin, H. (2018). High-cost sharing policies and non-urgent emergency department visits. International Journal of Health Care Quality Assurance (09526862), 31(7), 735-745. https://doi.org/10.1108/IJHCQA-05-2017-0089

Young-Harry, O. N., Dienye, P. O., & Diete-Spiff, K. O. (2015). Pattern of inappropriate cases presenting to the Accident and Emergency Department in a Nigeria Tertiary Hospital. South African Family Practice, 57(4), 252-258. https://doi.org/10.1080/20786190.2014.978114
